# A Behavioral Activation Digital Intervention Incorporating Gamification and Peer Support for Adolescent Depression in Rural South Africa: A Pilot Randomized Controlled Trial (the DoBAt Study)

**DOI:** 10.1016/j.jaacop.2025.06.009

**Published:** 2025-07-07

**Authors:** Bianca Moffett, Julia R. Pozuelo, Eustasius Musenge, Zamakhanya Makhanya, Heather A. O’Mahen, Michelle G. Craske, Alastair van Heerden, Crick Lund, Kate Orkin, Tholene Sodi, Sarah-Jayne Blakemore, Stephen Tollman, DoBAt & Ebikolwa Consortium, Princess Makhubela, Meriam Maritze, Nokhutula Mayindi, Ruvimbo Chiswo, Gabriele Chierchia, Emma J. Kilford, Sophie L. Fielmann, Munshi Sulaiman, Eugene Kinyanda, Mahreen Mahmud, Megan Davis, Christine Nabulumba, Doreen Sikoti, Joy Louise Gumikiriza-Onoria, Kathleen Kahn, Alan Stein

**Affiliations:** a University of the Witwatersrand, Johannesburg, South Africa; b South African Medical Research Council; c Harvard University, Cambridge, Massachusetts; d University of Oxford, Oxford, United Kingdom; e University of Cape Town, Cape Town, South Africa; f University of Exeter, Exeter, United Kingdom; g University of California, Los Angeles, California; h Human Sciences Research Council, Pietermaritzburg, South Africa; i King’s College London, London, United Kingdom; j University of Limpopo, Sovenga, South Africa; k University of Cambridge, Cambridge, United Kingdom; l Africa Health Research Institute, KwaZulu Natal, South Africa

**Keywords:** depression, adolescence, digital, gamification, peer support

## Abstract

**Objective:**

Effective and scalable interventions to address adolescent depression are urgently needed. This study evaluated the feasibility, acceptability, and preliminary efficacy of digitally delivered Behavioral Activation therapy.

**Method:**

A pilot randomized controlled trial was conducted in rural northeastern South Africa. Adolescents 15 to 19 years of age with mild-to-moderately severe depression on the Xitsonga version of the Patient Health Questionnaire—Adolescent Version (PHQ-A) were recruited from 11 high schools. Participants were randomly assigned (1:1) to receive the Kuamsha app, which delivers Behavioral Activation therapy through an interactive narrative game with telephone guidance from peer mentors or a control app. We used a mixed-methods design to assess the feasibility, acceptability, and preliminary efficacy of the Kuamsha app in reducing symptoms of depression compared to a digital control.

**Results:**

Between April and September 2022, a total of 195 adolescents were randomized. Primary outcome data were available on 195 adolescents (100%). In the Kuamsha group, 77 participants (80.2%) adhered to the treatment protocol (completed at least 4 of 6 app modules), indicating favorable engagement. In-depth interviews and questionnaire responses revealed high acceptability. Reductions in depressive symptoms were modest, with no significant between-group differences at 11 weeks (adjusted mean difference = −0.37, 95% CI = −1.96, 1.22) or 24 weeks (adjusted mean difference = −0.11, 95% CI = −1.70, 1.48). Exploratory analyses suggested greater efficacy among participants who met a cut-off for moderate baseline depression symptoms (PHQ-A ≥10) and greater app engagement.

**Conclusion:**

The Kuamsha app is a feasible and acceptable treatment for depression among adolescents in rural South Africa. Following adaptations, a larger trial is warranted to assess its effectiveness in reducing symptoms of depression.

**Diversity & Inclusion Statement:**

We worked to ensure sex and gender balance in the recruitment of human participants. We worked to ensure race, ethnic, and/or other types of diversity in the recruitment of human participants. We worked to ensure that the study questionnaires were prepared in an inclusive way. One or more of the authors of this paper self-identifies as a member of one or more historically underrepresented racial and/or ethnic groups in science. One or more of the authors of this paper self-identifies as a member of one or more historically underrepresented sexual and/or gender groups in science. One or more of the authors of this paper self-identifies as living with a disability. We actively worked to promote sex and gender balance in our author group. We actively worked to promote inclusion of historically underrepresented racial and/or ethnic groups in science in our author group. The author list of this paper includes contributors from the location and/or community where the research was conducted who participated in the data collection, design, analysis, and/or interpretation of the work.

## Plain language summary

This pilot trial (DoBAt) in rural South Africa tested a gamified mental health app named Kuamsha for adolescents with depression. The app, based on principles of behavioral activation and supported by peer mentors, was found to be feasible and acceptable in 195 adolescents with mild to moderate depression, though it was no more effective in reducing depressive symptoms than a control app. Researchers recommend refining the app to improve efficacy.

Adolescence, frequently thought of as 10 to 19 years but defined by some authors as 10 to 24 years,^1^ is a critical period during which individuals develop their self-identity, acquire the skills with which they navigate future challenges, and make decisions that can affect their long-term health, education, relationships, and employment prospects.^2^ It is also a period of heightened vulnerability to mental health conditions such as depression, which is the leading cause of disability worldwide and typically has its onset during mid-adolescence.^3^ Left untreated, depression interferes with the acquisition of key cognitive, social and emotional capacities, limiting young people’s ability to fulfill their potential.^4^ Depression is also a major risk factor for suicide and is associated with reduced educational attainment, risky behaviors, and substance abuse.^5^ Identification and treatment of depression among adolescents is thus a key public health concern and global development priority.^6^

More than 90% of the world’s adolescents live in low- and middle-income countries (LMICs),^3^ where less than 5% of those needing treatment for depression can access even minimally adequate care.^7^ Although effective psychological treatments exist, several barriers hamper the provision of and access to care. Insufficient public expenditure on mental health and a shortage of trained mental health personnel limit the supply of accessible and affordable mental health services.^8^ Although nonspecialist health care workers can deliver psychosocial interventions effectively, maintaining the quality of care requires ongoing training and supervision, particularly over the long term and when delivered at scale.^9^^,^^10^ Furthermore, prevailing stigma and low levels of mental health awareness continue to be important demand-side barriers to care.^11^

Digital delivery of psychological treatments offers a potential solution to overcome several of these barriers and to bridge the gap in adolescent mental health services in LMICs.^12^ In addition to providing a mechanism for the delivery of psychological treatments, digital interventions present an opportunity for screening, triage, and monitoring of mental health conditions, potentially extending the capacity to deliver mental health care and reducing the pressure on overstretched health care systems.^12^ Digital tools are appealing to adolescents, as they allow discreet access to care via personal devices and offer an accessible platform to target health education and to address mental health awareness.^13^ The flexibility and convenience of accessing care using a mobile device, coupled with reduced out-of-pocket expenses associated with travel to health care facilities, could further incentivize their use.^14^ Given the rapid global increase in mobile phone ownership and Internet access, empirical evidence to explore this potential is required.

Despite the rapid growth in research and development of digital psychological treatments, there has been limited focus on expanding access to care for underserved populations in socioeconomically disadvantaged areas.^15^ Although evidence supports the efficacy of digital psychological treatments for adults in both high-income contexts^16^ and LMICs,^17^ there is markedly less evidence for their effectiveness among school-aged adolescents (ie, 10-19 years of age), particularly in LMICs.^18^, ^19^, ^20^ A meta-analysis of 8 studies showed that smartphone apps can help to reduce depression symptoms in adolescents and young adults 10 to 35 years of age (Hedges *g* = 0.52, CI = 0.18-0.84, *k* = 8).^20^ Although the results were promising, the studies had some limitations, including high heterogeneity (*I*^*2*^ >70%), short follow-up, lack of active controls (*k* = 4), and a focus on young adults, with few studies including school-aged adolescents. In addition, none of these studies was conducted in an LMIC.

To the best of our knowledge, only 3 randomized controlled trials have assessed the efficacy of digital psychological treatments specifically designed to address depression among school-aged adolescents (10-19 years of age) in LMICs. Two of these studies, conducted in Kenya^21^ and Iran,^22^ assessed Web-based interventions delivered within school settings in group and individual formats, respectively. A third study assessed an app-based intervention, the “Coping Camp app,” also delivered within schools, which produced a small reduction in symptoms of depression (Cohen *d* = 0.11, *p* = .04) among Chinese school students compared to a nondigital control condition (attending moral education classes).^23^ A fourth study assessed the efficacy of a Web-based intervention using Behavioral Activation for adults in Indonesia and included a small subsample of adolescents.^24^

No studies have specifically evaluated digital psychological interventions tailored for adolescents in South Africa despite the high prevalence of mental health conditions. The point prevalence of depression^25^ and suicidal ideation^26^ among adolescents have been estimated at around 25% and 20%, respectively. Furthermore, children and adolescents in South Africa are often exposed to high levels of adversity.^27^ The need to scale up child and adolescent mental health services is acknowledged in national mental health policy and strategic plans,^28^ but little progress has been made in this regard, particularly in rural and under-resourced parts of the country. The Bushbuckridge area of rural Mpumalanga, where this study was conducted, was a former homeland area under Apartheid, and high levels of poverty, unemployment, and labor migration prevail. Development of infrastructure and services has been slow, and no formal child and adolescent mental health services currently exist.

The DoBAt Study in South Africa, in conjunction with its counterpart study in Uganda,^29^ was conducted to address the scarcity of scalable psychological treatments for adolescent depression in these countries. We adapted Behavioral Activation (BA) therapy for digital delivery in a rural African context, incorporating gamification and peer mentor support to enhance engagement.^30^ BA is an evidence-based psychological treatment for adolescent depression that has been shown to be effective in both clinical and subthreshold cases.^31^, ^32^, ^33^ It is simpler and more action oriented than Cognitive–Behavioral Therapy and has been demonstrated to be equally effective but less costly to deliver.^33^ BA can be delivered by lay providers and has been successfully implemented in low-resource and diverse cultural settings.^34^ BA delivered via online programs with minimal support from lay counselors or peer mentors has been shown to be as effective as in-person delivery,^35^ further supporting its feasibility in this study. This adaptability makes BA particularly well suited for a gamified digital intervention, ensuring accessibility and engagement for adolescents.

The Kuamsha app was developed through an iterative co-design process with more than 160 adolescents and other stakeholders, described in detail elsewhere.^36^ Briefly, the app is an interactive narrative game comprising 2 stories, each containing 6 tailored modules that incorporate BA’s core principles. The app is supported by telephone guidance from trained peer mentors.^37^

This study reports our findings regarding the 2 co–primary objectives of the DoBAt Study. The first objective was to assess the feasibility and acceptability of the Kuamsha app as a treatment for depression among adolescents in rural South Africa. The second objective was to assess the preliminary efficacy of the intervention in reducing depressive symptoms compared to that of an app that controlled for digital use and featured locally produced wildlife videos.

## Method

### Study Design

The DoBAt study was a 2-arm, parallel, individual-level, randomized controlled pilot trial and feasibility study (N = 195) conducted in the study area of the SAMRC/Wits Rural Public Health and Health Transitions Research Unit that runs the Agincourt Health and socio-Demographic Surveillance Site (HDSS) in Mpumalanga province, northeastern South Africa. Ethical approval was provided by the University of the Witwatersrand Human Research Ethics Committee (MED20-05-011) and the Oxford Tropical Research Ethics Committee (OxTREC 34-20). Further details are available in the published protocol.^38^

### Participants

Recruitment occurred over 5 months at 11 schools within the study area. To be eligible for inclusion, adolescents had to meet the following criteria: (1) to be between 15 and 19 years of age and in grades 9 to 11; (2) to exhibit symptoms of mild to moderately severe depression, indicated by a score between 5 and 19 on the Xitsonga version of the Patient Health Questionnaire—Adolescent Version (PHQ-A); (3) to demonstrate sufficient reading ability in the local language (Xitsonga) to use the Kuamsha app; (4) to intend to continue living in the study area for 12 weeks after the baseline assessment; and (5) to provide written informed assent/consent to participate in the study, as well as parent/guardian consent if under 18 years of age.

Participants were excluded if they met the following exclusion criteria: (1) had symptoms of severe depression (PHQ-A score >19); (2) reported current suicidal ideation with specific plans and means identified; (3) were receiving psychological treatment for a mental health condition at the time of enrolment; (4) had been hospitalized for at least 5 days because of severe psychiatric, life-threatening, or other serious medical illness; (5) had a history of bipolar mood disorder, schizophrenia, or other psychotic disorder; or (6) lacked the capacity to consent to treatment or research participation or to use the app. Excluded participants were assessed by the Risk Management Team and referred to local health and social care services, with initial care provided by the team when local services were delayed.

### Randomization and Masking

Participants were randomly assigned to the intervention or control arm with a 1:1 allocation ratio using a computerized minimization algorithm, balanced by sex (male or female participants) and severity of depressive symptoms (<10 or ≥10 on the PHQ-A). The minimization algorithm was generated by the Centre for Healthcare Randomized Trials (CHaRT) at the University of Aberdeen. Participants were allocated using CHaRT’s online software, and the trial manager oversaw the provision of the smartphones with the treatment or control app pre-installed to participants as per assignment. Allocation concealment (blinding) was applied to all fieldworkers conducting quantitative outcomes assessments, as well as the trial statistician (EM) who conducted the analysis of the preliminary efficacy of the intervention.

### Procedures

The trial consisted of an 11-week intervention phase (including 6 modules/sessions), and participants were followed for a further 13 weeks after completing the intervention (ie, a total of 24 weeks).

To standardize conditions across treatment groups, all participants received a basic smartphone (Samsung Galaxy A2 Core) with the assigned app pre-installed, both of which function offline (ie, without mobile Internet data or WiFi connection). To enable the upload of app-usage data and to respond to online surveys, 200 MB of mobile Internet data were provided at 6 different time points (0, 2.5, 5, 7.5, 11, and 24 weeks), totaling 1.2 GB over the study period. Depressive symptoms were monitored using the PHQ-A via brief online surveys sent via text message at these same time points.

Adolescents in the treatment group received the Kuamsha app, a gamified app that delivers BA therapy using storytelling techniques and game design elements. The app consists of 2 stories, the Football Match and the Song Contest, each containing 6 tailored modules with interactive components. Each module takes approximately 15 to 20 minutes to complete and covers different BA skills. After the first module, participants are asked to think of a realistic and achievable goal to address over the intervention period. For all consecutive modules, users are encouraged to work on weekly activities related to the module principle that aligns with this goal and to report their progress and mood as they complete them. The name “Kuamsha,” which means “activate” in Swahili (a language widely spoken in eastern and southern Africa), was selected by adolescents in South Africa and Uganda during the co-design process. Further details of the Kuamsha app are available in an associated publication^36^ and in Table S1, available online.

The Kuamsha app, which is the core component of the treatment, is supported by up to 6 weekly, 15- to 20-minute phone calls from trained peer mentors. Peer mentors were Xitsonga-speaking students or recent bachelor’s-level graduates from the department of psychology or social work at an accredited South African university. They were trained to provide brief structured support to with the following objectives: (1) to motivate adolescents to use the app; (2) to provide clarification around any aspects of the module that adolescents struggled to understand; and (3) to help adolescents think about how they could implement the homework activities, that is, apply the principles of BA to their daily lives. Notably, the peer mentor calls did not introduce any new content but instead clarified the app content. We thus define the Kuamsha treatment as a “low-intensity” psychological treatment^37^ and consider 1 dose of treatment to be 1 session of the app completed. Further details of the training and supervision of peer mentors will be provided in a separate publication.

Participants in the control group received the “Kuchunguza” app (meaning “explore” in Swahili), which was specifically developed for this study to provide an appropriate digital placebo that did not provide elements of BA. The Kuchunguza app contains six 15- to 20-minute videos from WildEarth-SafariLive, a locally produced wildlife series. As noted above, control participants also received active symptom monitoring via online surveys.

Surveys were conducted in person at participants’ households at baseline and at 11 weeks and 24 weeks after baseline using a combination of Computer-Assisted Personal Interviews (CAPI) and Audio-Computer Assisted Self-Interviews (ACASI). Participants received a snack on completion of in-person surveys. The postintervention acceptability survey was conducted telephonically by a separate fieldwork team. App engagement metrics (including treatment adherence) were captured on an online database as participants used the app.

In-depth interviews to assess the feasibility, acceptability, and appropriateness of the intervention were conducted with 21 adolescents and their parents after completing the intervention. They were selected using a random sample of participants from both the treatment and control groups (2:1 ratio) and were stratified by their level of engagement with the app to elicit a variety of perspectives. Experienced bilingual (Xitsonga- and English-speaking) qualitative fieldworkers who lived and worked in the study area conducted the interviews with participants in Xitsonga between August 2022 and January 2023. Interviews were audio recorded, translated into English, and transcribed by the same fieldworkers who conducted the interviews using an intelligent (ie, naturalized) verbatim approach.

### Outcomes

This study had the following 2 co–primary objectives.

### Co–primary Objective 1: Feasibility and Acceptability

We used an explanatory-sequential mixed-methods design to assess feasibility and acceptability. Progression from the pilot to a larger trial was guided by predefined trial progression criteria, which were based on feasibility measures and categorized into 3 zones: “green” for proceed, “amber” for advance with caution and modifications, and “red” for halting progression (Table S2, available online).

#### Feasibility

1) Recruitment and retention rates. We assessed the proportion of eligible participants enrolled in the study and retained at 11 weeks. Enrollment rates of ≥60% were considered to fall into the “green” zone; rates of <60% and ≥40% into the “amber” zone; and rates of <40% in the “red” zone. Retention rates of ≥90% were considered to fall into the “green” zone; rates between <60% and ≥50% into the “amber” zone; and rates of <50% in the “red” zone.
2) Assessment completion rates. We examined the completion rates across all study assessments to evaluate their feasibility, but no specific progression criteria were prespecified.
3) Treatment adherence rates and app engagement metrics. Treatment adherence was defined *a priori* as having opened at least 4 of 6 of the app modules and completed 3 of 6 calls with the peer mentor (excluding the introductory call). Adherence was included in our trial progression criteria, which delineated “green” for adherence levels of ≥70%, “amber” for levels between <70% and ≥50%, and “red” for levels of <50%.

#### Acceptability

An acceptability questionnaire was administered that contained 3 measures: Acceptability of Intervention Measure (AIM), Intervention Appropriateness Measure (IAM), and Feasibility of Intervention Measure (FIM).^39^ In addition, in-depth interviews were conducted to explore participants’ and parents’ perspectives on the feasibility, acceptability, and appropriateness of the app.

We also assessed the fidelity of delivery of the peer mentor calls by collecting data on the adherence and competence of trained peer mentors. Adherence was measured by the proportion of phone calls meeting at least 90% of the criteria from the training protocol, rated independently by 2 raters across various domains (eg, professionalism, cultural sensitivity). Competence was evaluated using a competency assessment test, scored out of 100, including written and role-playing components, scored by the peer mentor supervisor.

### Co–primary Objective 2: Preliminary Efficacy

The preliminary efficacy of the intervention in reducing symptoms of depression compared to the control condition was assessed using the PHQ-A. The PHQ-A is a widely used and well-established measure of adolescent depressive symptoms over the past 2 weeks.^40^ We used the Xitsonga version of the PHQ-A to assess depressive symptoms at 6 assessment points (screening and weeks 2.5, 5, 7.5, 11, and 24).

### Assessment of Safety and Adverse Events

Participants who developed severe depression (PHQ-A score >19) or suicidal ideation (PHQ-A ninth-item score ≥1) during the trial were assessed by the study Risk Management Team and referred to local health and social services in accordance with a predefined study Risk Management Protocol. These participants were retained in the study.

### Sample Size Determination

Statistical power was calculated to detect differences between 2 independent groups in a 2-sided test with an α of 0.05 and a power of 1–β = 0.80 for an effect size (Cohen *d*) of 0.45. This choice was informed by the existing literature, and we factored in a 25% attrition rate. Given these assumptions, we aimed to recruit 200 participants.^38^

### Data Analysis

Quantitative data were analyzed using Stata version 17.0.^41^ Outcomes were assessed on an intention-to-treat basis. We used descriptive analyses to explore patterns in the data, followed by inferential statistics involving univariate and multivariable models. A 2-tailed *p* value of <.05 was considered statistically significant in the inferential analyses.

Analysis of the efficacy of the intervention on depressive symptoms was based on the PHQ-A score at 11 weeks (primary endpoint) and 24 weeks (secondary endpoint) using linear regression adjusted for age, sex, household asset index, and PHQ-A score at screening. We used robust standard errors to allow for the presence of heteroskedasticity. As a secondary analysis, we used the repeated measurements of the PHQ-A throughout the trial (up to 6 times per participant) to evaluate the treatment effects over time using generalized estimating equations. An independent Data and Safety Monitoring Board and a Trial Steering Committee provided trial oversight, and the trial was prospectively registered with the Pan African Clinical Trials Registry (PACTR202206574814636).

Qualitative interviews were analyzed using Thematic Analysis^42^ with the use of NVivo software to facilitate the analysis.

This study adhered to the Consolidated Standards of Reporting Trials (CONSORT) extension for pilot and feasibility trials (Table S3, available online).

## Results

**Figure 1:**
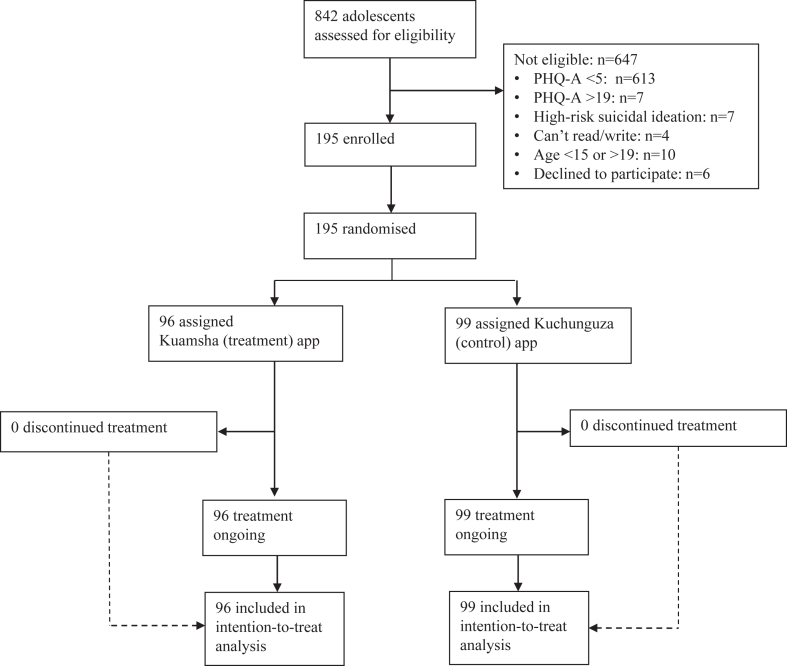
Trial Profile ***Note:****PHQ-A = 9-item Patient Health Questionairre*—*Adolescent Version*.

Participants were enrolled from April 5 to September 5, 2022 (Figure 1). A total of 842 adolescents at 11 high schools within the study area completed the screening survey. Of these, 229 participants (27.2%) showed some symptoms of depression (PHQ-A≥5), and 77 (9.1%) had PHQ-A scores of ≥10. In total, 14 screened adolescents (1.7%) were not eligible for inclusion because of severe depression and/or high-risk suicidal ideation, and were referred to local services. Six adolescents declined to participate because of concerns that the study would interfere with their schoolwork. Other reasons for ineligibility are given in Figure 1. A total of 195 adolescents were enrolled and randomized.

Baseline demographic characteristics and PHQ-A scores are shown in Table 1.^43^^,^^44^ The median age of study participants was 16 years (interquartile range [IQR] = 15-17), and approximately two-thirds of the sample self-identified as female. There was 1 protocol deviation, whereby a participant in grade 8 was recruited. Most households were relatively poor, with around 10% living below the international extreme poverty line of $1.90 per day, and almost three-fourths (73.3%) were food insecure. Most participants (69.7%) had mild depressive symptoms (5≤PHQ-A<10).

**Table 1:** Baseline Characteristics of Trial Participants

	Total (N = 195)	Control (n = 99)	Treatment (n = 96)
Age, y, mean (SD)	16.14 (1.05)	16.26 (1.14)	16.02 (0.94)
Sex^a^			
Female, n (%)	132 (67.69)	67 (67.68)	65 (67.71)
Male, n (%)	63 (32.31)	32 (32.32)	31 (32.29)
Married, n (%)	7 (3.59)	5 (5.05)	2 (2.08)
Lost a parent, n (%)	66 (33.85)	35 (35.35)	31 (32.29)
Has children, n (%)	10 (5.68)	7 (7.87)	3 (3.45)
Grade enrolled at screening			
Grade 8, n (%)	1 (0.51)	1 (1.01)	0 (0.00)
Grade 9, n (%)	25 (12.82)	10 (10.10)	15 (15.62)
Grade 10, n (%)	85 (43.59)	40 (40.40)	45 (46.88)
Grade 11, n (%)	84 (43.08)	48 (48.48)	36 (37.50)
Did any work in past 7 days, n (%)	38 (19.69)	20 (20.62)	18 (18.75)
Household asset index^b^	39.98 (14.57)	41.72 (14.94)	38.22 (14.04)
Food insecure,^c^ n (%)	140 (73.30)	69 (71.88)	71 (74.74)
Caregiver’s years of education^d^	8.21 (4.60)	8.59 (4.54)	7.84 (4.66)
PHQ-A score, mean (SD)	8.51 (3.43)	8.40 (3.55)	8.62 (3.31)
Depression symptom category			
Mild (5≤PHQ-A<10)	136 (69.74)	69 (69.70)	67 (69.79)
Moderate (10≤PHQ-A<15)	45 (23.08)	21 (21.21)	24 (25.00)
Moderately severe (15≤PHQ-A<19)	14 (7.18)	9 (9.09)	5 (5.21)

PHQ-A = 9-item Patient Health Questionnaire—Adolescent Version.

Note: Data are n (%), or mean (SD).

a Sex is based on participant self-report.

b Household asset index was measured using the Simple Poverty Scorecard Poverty-Assessment Tool South Africa.^43^ For reference, scores between 38 and 40 correspond to a 9.8% likelihood of living below the international extreme poverty line of $1.90 per day (PPP, 2011 prices).

c Food insecurity was measured using the 6-item Food Security Module.^44^

d Caregiver refers to an adult with joint or sole caring responsibility in the index adolescent’s household.

Figure 1 shows high recruitment and retention rates, with 97% of eligible participants successfully recruited and all 195 enrolled participants (100%) completing the 11-week primary endpoint. This retention rate places the study well within the “green” zone according to our trial progression criteria. In addition, 194 participants (99%) completed the acceptability survey (via telephone), and 192 (98.5%) completed the 24-week follow-up survey (in person). Completion rates for the online symptom monitoring sent via SMS were substantially lower, averaging 45.1% across all participants. The control group had higher completion rates on average, with significant differences observed at week 2.5 (*p* = .005) and week 7.5 (*p* = .017), but no significant differences at week 5, as detailed in Table S4, available online.

Table 2 shows the app engagement metrics for the treatment and control groups. Among participants in the treatment group, 77 (80.2%) completed 4 or more modules, and 74 (78.7%) completed 3 or more phone calls with their peer mentor (excluding the introductory call, which all participants had). Both treatment adherence metrics exceeded the minimum 70% adherence rate, placing them within the “green” zone for trial progression. In addition, 61 participants (63.54%) completed the recommended 6 app modules, and 52 (55.3%) completed all 6 calls with their peer mentor. In total, 16 participants (16.67%) did not complete any modules, and the number of phone calls completed varied, with a median of 6 calls (IQR = 3-6) and 11 participants (11.7%) completing only 1 call.

**Table 2:** Feasibility and Acceptability Outcomes

	Control (n = 99)	Treatment (n = 96)	*p*
Feasibility			
Participants with data, n (%)	61 (62)	96 (100)	
Treatment adherence			
Opened ≥4 app modules, n (%)	16 (26.22)	77 (80.2)	<.0001
Had ≥3 phone calls with peer mentors, n (%)	NA	76 (79.2)	NA
App engagement metrics			
Log-ins, mean (SD)	NA	29.68 (30.18)	NA
Modules opened, mean (SD)	2.41 (1.84)	16.29 (17.46)	<.0001
Modules completed, mean (SD)	1 (1.46)	12.21 (14.70)	<.0001
Total time spent on app (h:min), mean (SD)	00:26 (00:34)	3:34 (3:52)	<.0001
Set up weekly activities, mean (SD)	NA	5.63 (4.33)	NA
Completed weekly activities, mean (SD)	NA	55.93 (125.39)	NA
Peer mentor engagement metrics^a^			
Participants with data, n (%)	NA	94 (97.9)	NA
Phone call duration (min), mean (SD)	NA	16.59 (4.85)	NA
Time between phone calls, days, mean (SD)	NA	9.81 (8.74)	NA
Participants who completed all phone calls, n (%)	NA	52 (55.3)	NA
Acceptability			
Acceptability of using the app			
AIM score, mean (SD)	3.98 (0.75)	4.22 (0.71)	.024
IAM score, mean (SD)	3.89 (0.80)	4.22 (0.64)	.002
FIM score, mean (SD)	3.96 (0.69)	4.09 (0.63)	.16
Acceptability of the peer mentor calls			
AIM score, mean (SD)	NA	4.44 (0.50)	NA
IAM score, mean (SD)	NA	4.20 (0.67)	NA
FIM score, mean (SD)	NA	4.20 (0.64)	NA

Note: Data are n (%) or mean (SD). AIM = Acceptability of Intervention Measure; FIM = Feasibility of Intervention Measure; IAM = Intervention Appropriateness Measure; NA = not applicable.

a Peer mentor engagement metrics are based on recordings of 512 calls.

On average, the Kuamsha app was launched 29.68 times per participant, and users spent approximately 3.5 hours on it over the intervention period. The average number of modules completed was 12.21, and 37 participants (38.54%) completed both stories, totaling 12 modules. A third of the participants went through the stories more than once, which explains why the average exceeds the number of modules in the app. Initially, many participants went through the episodes very quickly, and peer mentors later guided them to restart the stories to ensure that sufficient time was dedicated to weekly activities. Notably a quarter of participants continued using the app post intervention. We found no significant difference in engagement metrics by depressive symptoms (Table S5, available online).

Participants identified various goals during the intervention. The most frequently chosen goals included reading every night, getting better grades at school, helping at home, maintaining a regular bedtime, exercising, and making new friends. Some goals lacked specificity, measurability, or time constraints (eg, “to persevere,” “dance to my favorite music,” “do my best”), and peer mentors were trained to assist in refining these during weekly calls.

Overall, we found a moderate positive correlation between the frequency of phone calls with peer mentors and app engagement. Those who engaged in more calls were more likely to log in to the app (standardized β = 0.23, *p* < .05), to open and complete app modules (standardized β between 0.21 and 0.29, *p* < .05), and to complete more homework activities (standardized β between 0.21 and 0.25, *p* < .05).

Table 2 also shows the app engagement metrics for the control group. Data are available for only 61 participants (62%) because of a device-linking issue. There were no significant differences in most sociodemographic characteristics or in depression severity between participants with and without data, except for sex (whereby male participants were more likely to have missing data, *p* = .012) (Table S6, available online). Control participants exhibited lower engagement levels, opening an average of 2.41 videos (SD = 1.84) and completing 1 video (SD = 1.46). The average time spent in the app was 26 minutes. There was no significant difference in app use by depression status (Table S5, available online).

Quantitative findings regarding the acceptability

Acceptability (Acceptability of Intervention Measure), appropriateness (Intervention Appropriateness Measure), and feasibility (Feasibility of Intervention Measure) of the treatment and control apps are also displayed in Table 2. Participants in the treatment group rated the Kuamsha app as significantly more acceptable (*p* = .024) and appropriate (*p* = .002) than participants in the control group rated the wildlife app, whereas feasibility perceptions were comparable between the 2 groups (*p* = .16). The acceptability of peer mentor calls among participants in the treatment group was positively endorsed.

Qualitative findings from interviews conducted with 14 adolescents in the treatment group and their parents extended our understanding of the feasibility and acceptability of the Kuamsha app and provided insights into the barriers and facilitators to user engagement. In addition, interviews with 7 participants in the control group provided insights into engagement with the control wildlife app.

Most participants in the treatment group reported that they liked the Kuamsha app and found it easy to use. Furthermore, several participants described valuable lessons that they had learned and noted positive changes in their thoughts, feelings, or behaviors.

“The app helped me to assess what I want in life. It taught me about how to choose my career, the things I want to achieve while still at school, and the things that I want to do in my life.” (Kuamsha participant (P) 10)

“I have learnt how to control my anger, be able to communicate with others and understand them, and I have also learnt what I need to do when I am feeling low or thinking about bad things.” (Kuamsha P3)

Participants in the treatment group identified several app features that facilitated engagement, including in-app points and games within the stories. They also found the stories and characters relatable to their own lives. Several adolescents reported that their peer mentor helped them to understand and to use the Kuamsha app.

“It was good having a peer mentor because when she calls, she would ask what I have learnt on that current episode and also help me understand some of the things I didn’t understand.” (Kuamsha P9)

Some adolescents’ narratives of their engagement with their peer mentor suggest that the peer mentor’s role extended beyond supporting app adherence.

“The person who was calling me engaged herself in many things. She talked about school issues, domestic violence, and the behavior itself. I was open to talk to her and she was open talking to me too. I think the way she spoke to me was the most helpful thing ever.” (Kuamsha P5)

Adolescents also reported barriers to engaging with the Kuamsha program, notably the following: (1) a desire for more content and modules; (2) finding that the app required too much reading; (3) experiencing technical issues with the app or phone; and (4) difficulty finding a mutually convenient time to talk to their peer mentor.

“I think they must add to them (the modules). They are not enough, and if you completed them, you have to start again and you will do one thing.” (Kuamsha P2)

Furthermore, a few participants reported that they would have liked to receive some in-person support or social intervention, such as a facilitated family discussion.

“I had a problem with my maternal family and I was staying with them. I think it would have been better for me if maybe someone came to talk to them.” (Kuamsha P7)

Some participants in the control group reported that the Kuchunguza (ie, control) app was beneficial. They also appreciated receiving a smartphone and valued being asked about their mental health in a private and confidential manner during assessments.

“I think the app is very good because for me whenever I felt sad, I would just watch the videos and it would calm me down.” (Kuchunguza P5)

Regarding the fidelity of intervention delivery, peer mentors attained an average competency score of 62.96% (SD = 20.19) on the post-training assessment, indicating a moderate level of proficiency before starting with study participants. Those with scores of <50% were required to retake the test until they achieved the minimum satisfactory score. Independent raters evaluated and rated 40 of the 421 calls made by peer mentors. Of these, 65% met an adherence level of 80% or above, whereas 30% of calls achieved the higher adherence criterion of 90% or more. This suggests a low to moderate overall adherence to the training protocol, with some variability in performance among mentors.

Table 3 shows the results of primary and secondary analyses regarding the preliminary efficacy of the intervention in reducing symptoms of depression. By the end of the intervention (week 11), participants in both arms experienced a modest reduction in depressive symptoms. Notably, the treatment group displayed a slightly more substantial reduction in depressive symptoms, in line with our expected direction of change. However, although there are some indications of greater improvement in PHQ-A scores in the treatment group compared to the control group, the differences observed did not reach statistical significance across primary and secondary outcomes.

**Table 3:** Preliminary Efficacy

	Treatment group (n = 96)	Control group (n = 99)	Cohen *d* (95% CI)	Adjusted mean difference (95% CI)	*p*
**Primary**					
Mean PHQ-A score at 11 wk (SD)^a^	7.13 (5.56)	7.55 (5.66)	−0.07 (−0.36, 0.21)	−0.37 (−1.96, 1.22)	.646
**Secondary**					
Mean PHQ-A score at 24 wk (SD)^a^	7.31 (5.24)	7.34 (6.07)	−0.01 (−0.29, 0.28)	−0.11 (−1.70, 1.48)	.894
Remission^c^: PHQ-A score <5 at 11 wk, n (%)^b^	35 (36.46%)	41 (41.41%)	−0.10 (−0.38, 0.18)	−0.04 (−0.18, 0.09)	.516
Recovery^d^: PHQ-A score <5 at 11 and 24 wk, n (%)^b^	23 (23.96%)	25 (25.25%)	−0.03 (−0.31, 0.25)	−0.00 (−0.12, 0.12)	.992

Note: Robust standard errors were used to allow for the presence of heteroskedasticity. PHQ-A = 9-item Patient Health Questionnaire—Adolescent Version.

a Linear regression adjusted for age, sex, Household Assets Index, and PHQ-A score at screening.

b Logit regression adjusted for age, sex, Household Assets Index, and PHQ-A score at screening.

c Remission is a binary variable equal to 1 if the PHQ-A score is <5 at week 11.

d Recovery is a binary variable equal to 1 if the PHQ-A score is <5 at weeks 11 and 24.

We conducted 3 additional post hoc analyses. First, we examined treatment effects on participants with moderate to moderately severe depression (10≤PHQ-A<19) at the time of enrollment (n = 59) (Table S7, available online). Although we did not observe any between-group difference at our primary endpoint (adjusted mean difference [AMD] = −0.40, CI = −3.56, 2.75, Cohen *d* = −0.01), there was a nonsignificant trend favoring the treatment group at the 24-week follow-up (AMD = −1.62, CI = −4.76, 1.51, Cohen *d* = −0.25).

Second, we explored treatment effects among highly engaged adolescents (defined as those in the top third in terms of their app usage time, n=52) (Table S8, available online). The results of these analyses suggest nonsignificant between-group differences favoring the treatment group at 11 weeks (AMD = −1.47, CI = −4.48, 1.53, Cohen *d* = −0.28) and 24 weeks (AMD = −1.26, CI = −4.91, 2.38, Cohen *d* = −0.25).

**Figure 2:**
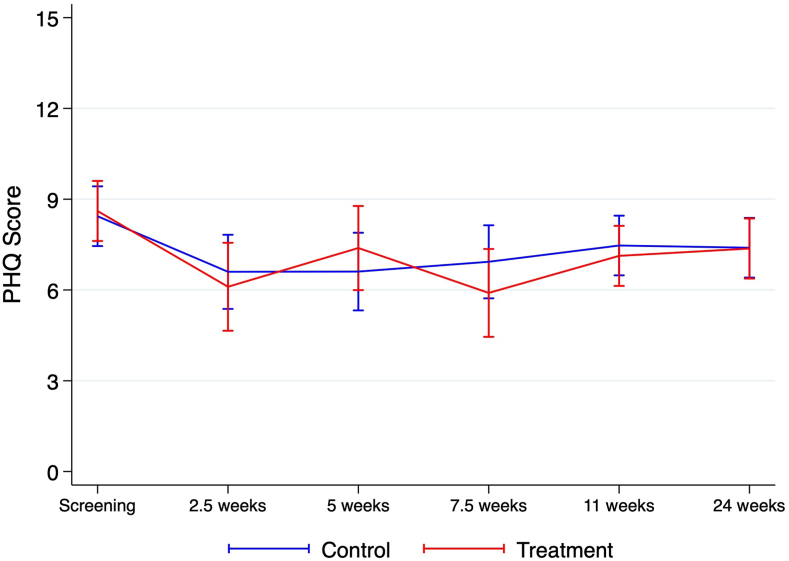
Observed Patient Health Questionnaire (PHQ) Scores Over Time by Treatment Arm (With 95% Confidence Intervals)

Third, a mixed-effects model was used to assess the effect of the intervention on depressive symptoms over time, adjusting for the same covariates as in our primary inference. The model showed a statistically significant decrease in PHQ-A scores from baseline (Table S9, available online). However, the rate of change in PHQ-A scores did not significantly differ between the treatment and control groups (Figure 2; Table S9, available online).

There were no significant differences in adverse events or risk management interventions among participants in the treatment and control groups (Table S10 and Table S11, available online).

## Discussion

The results of this study support the feasibility and acceptability of the Kuamsha app, supplemented by peer mentor calls, as a treatment for depression among adolescents in rural South Africa. We observed high recruitment (97%), retention (100%), and app adherence (80.2%) rates among participants in the treatment group, indicating favorable uptake and engagement with the digital intervention. Qualitative findings further supported these results, showing that most adolescents perceived the Kuamsha app and peer mentor calls to be an acceptable, feasible, and appropriate treatment for depression.

Our second co–primary objective was to assess the preliminary efficacy of the intervention in reducing symptoms of depression compared to the control. Although we observed a modest reduction in depressive symptoms among participants in both the treatment and control groups, there was no significant between-group difference at our primary endpoint (11 weeks) or follow-up assessment (24 weeks). Three key reasons are likely to explain this result.

First, we observed relatively high engagement with our control app, and a small, nonsignificant reduction in depressive symptoms from baseline to follow-up (*d* = 0.18, *p* = .17).

Several adolescents described the control app as “calming” or helpful in taking their minds off their problems. Although there is evidence that exposure to nature and positive imagery can improve mood,^45^^,^^46^ our findings suggest that these effects were weak and unlikely to explain symptom changes in our study. Furthermore, participants in the control group also reported benefiting from receiving a smartphone and being asked about their mental health during the study assessments in a private and confidential manner. These factors, together with the active symptom monitoring and risk management interventions in both study arms, may have further minimized between-group differences. These findings are aligned with meta-analyses of digital psychological interventions among both adult^19^ and adolescent^47^ populations, which indicate smaller effect sizes when active controls are used as opposed to treatment as usual or waitlisted controls.

Second, two-thirds of our study sample had mild depressive symptoms (5≤PHQ-A<10) at enrollment, and subgroup analyses suggest that the Kuamsha app may have had greater efficacy among participants with moderate to moderately severe depressive symptoms (10≤PHQ-A<19). Although we cannot rule out the possibility that this finding is due to chance alone, a further larger trial should be adequately powered to assess treatment effects among this subgroup experiencing moderate to moderately severe depressive symptoms. This is supported by recent meta-analyses indicating that digital psychological treatments tend to have larger effects among participants with greater baseline symptom severity.^48^^,^^49^

Third, although the Kuamsha app represents a promising minimum viable digital product, our pilot trial provides important insights for enhancing engagement and efficacy. Adolescents particularly liked the game-design elements and that the stories and characters were relatable to their own lives—features that reflect the extensive process of user-centered co-design.^36^ Further adaptations could include improving user experience, developing more content, refining the stories to make Behavioral Activation therapy principles easier to understand, simplifying the weekly activity reporting, and offering alternatives to text (such as a story-video format).

Beyond these factors, our findings highlight considerable heterogeneity in engagement. Some adolescents completed most of the modules, whereas others engaged minimally. Similarly, although some participants highly valued the peer mentor support, others preferred to use the app independently and at their own pace. The moderate positive correlation between call completion and app engagement suggests that peer support may enhance adherence; however, it remains unclear whether symptom reductions were driven by the app alone, the calls, or their combined effect. Moreover, both adolescents and peer mentors indicated that some participants would have benefited from a more extended or flexible support model.

These findings have important implications for scalability and cost-effectiveness. Although the Kuamsha app could be delivered as a standalone intervention, incorporating peer mentor support may improve engagement and treatment effects for certain adolescents. A more personalized approach—whereby the intensity and duration of peer support are adjusted based on individual needs—may optimize effectiveness while maintaining feasibility. Prior research suggests that individual characteristics, such as baseline symptom severity and engagement patterns, can help to predict who will benefit most from additional support.^50^, ^51^, ^52^ A larger, adequately powered trial with a 3-arm design—comparing (1) the Kuamsha app alone, (2) the Kuamsha app + peer mentor support, and (c) an active digital control—would allow for a more nuanced understanding of what works, for whom, and how, helping to tailor support to adolescents who may benefit most from additional intervention.

In summary, our findings on the feasibility and acceptability of the Kuamsha app are important, given that user engagement remains a significant barrier to the effectiveness of digital psychological treatments and that few such interventions have been specifically developed to target adolescents in LMIC contexts.^48^ The Kuamsha app is a novel innovation, combining narrative game-design features with low-intensity telephonic support by trained peer mentors to deliver Behavioral Activation therapy in an engaging digital format. Key strengths of this study include a rigorous co-design process, robust study design with an active control, mixed-methods approach integrating both quantitative and qualitative data, comprehensive app engagement metrics, a relatively large sample size for a pilot study, high follow-up rates, and the inclusion of a 6-month long-term follow-up. Although between-group differences were not significant, publishing these findings is important for building an accurate evidence base, particularly given the reliance on waitlist controls in many digital mental health trials. In addition, our focus on adolescents in a rural, socioeconomically disadvantaged African setting—a neglected population in research—addresses the unique challenges of reaching young people in underserved areas and implementing a complex intervention. Together, these factors support the feasibility and utility of a further, larger trial and provide critical insights for refining the intervention and study design.

A number of limitations are noted. Firstly, the study was underpowered to detect an effect of the intervention among adolescents with moderate to moderately severe symptoms (10≤PHQ-A<19). Secondly, this was a complex intervention, with multiple interacting components (app, peer mentor calls, and regular symptom monitoring), therefore making it difficult to determine which elements contributed most to the observed outcomes. The intervention arm received both the Kuamsha app and 6 peer mentor calls, effectively increasing the total intervention engagement compared to the control arm, which only received six app-based modules. While the peer mentor calls were intended to support adherence rather than introduce new therapeutic content, they may have nonetheless contributed to engagement and potential treatment effects. Future research should isolate the impact of these components to better understand their individual and combined effects. Thirdly, this study was conducted in rural South Africa, where adolescent mental health resources are severely underdeveloped, although access to social protection, healthcare and education exceeds that of many other African countries - findings from this study should be triangulated with those from our sister study conducted in Uganda. Finally, we chose to give adolescents in both the treatment and control groups a basic smartphone and small amounts of data to standardize conditions across arms, thus implying that the feasibility of the digital intervention is contingent on adolescents having access to a smartphone and data.

In conclusion, the Kuamsha app, supported with low-intensity telephonic guidance from peer mentors, presents a novel and promising approach to addressing depression among adolescents in rural South Africa. The intervention should be adapted to optimise engagement and effectiveness. Following adaptations, a larger trial is warranted to assess the effectiveness of the intervention as a treatment for depression. Given the potential scalability, considerations around cost-effectiveness and implementation should be incorporated into the study design.

## CRediT authorship contribution statement

**Bianca Moffett:** Writing – original draft, Conceptualization, Writing – review & editing, Data curation, Methodology, Formal analysis, Project administration. **Julia R. Pozuelo:** Funding acquisition, Project administration, Writing – review & editing, Data curation, Writing – original draft, Conceptualization, Formal analysis. **Eustasius Musenge:** Methodology, Writing – review & editing, Formal analysis. **Zamakhanya Makhanya:** Project administration, Data curation, Writing – review & editing. **Heather A. O’Mahen:** Writing – review & editing, Conceptualization, Funding acquisition, Methodology, Investigation, Supervision. **Michelle G. Craske:** Supervision, Writing – review & editing, Conceptualization, Investigation, Funding acquisition, Methodology. **Alastair van Heerden:** Writing – review & editing, Funding acquisition, Conceptualization, Methodology. **Crick Lund:** Writing – review & editing, Conceptualization, Investigation, Funding acquisition, Supervision. **Kate Orkin:** Writing – review & editing, Conceptualization, Investigation, Funding acquisition, Methodology. **Tholene Sodi:** Writing – review & editing, Conceptualization, Investigation, Funding acquisition, Supervision. **Sarah-Jayne Blakemore:** Writing – review & editing, Conceptualization, Investigation, Funding acquisition, Methodology. **Stephen Tollman:** Resources, Supervision, Conceptualization, Investigation, Writing – review & editing, Funding acquisition, Methodology. **Kathleen Kahn:** Supervision, Conceptualization, Writing – review & editing, Funding acquisition, Methodology, Investigation, Resources. **Alan Stein:** Investigation, Methodology, Validation, Conceptualization, Supervision, Writing – review & editing, Funding acquisition.
