# Supplementary material for: A Behavioral Activation Digital Intervention Incorporating Gamification and Peer Support for Adolescent Depression in Rural South Africa: A Pilot Randomized Controlled Trial (the DoBAt Study)
Source: JAACAP Open. 2025 Jul 7;3(4):1270–83. doi: 10.1016/j.jaacop.2025.06.009 (PMC12684694; doi:10.1016/j.jaacop.2025.06.009)
Supplement: Supplemental Tables [file mmc1.docx]

**SUPPLEMENTARY MATERIALS**

Table of Contents

[Table S1. KUAMSHA APP’S MAIN COMPONENTS 2](#_Toc198592755)

[Table S2. TRIAL PROGRESSION CRITERIA 6](#_Toc198592756)

[Table S3. CONSORT EXTENSION FOR PILOT AND FEASIBILITY STUDIES CHECKLIST 7](#_Toc198592757)

[Table S4. COMPLETION RATES OF STUDY ASSESSMENTS 9](#_Toc198592758)

[Table S5. FEASIBILITY OUTCOMES BY DEPRESSION STATUS 10](#_Toc198592759)

[Table S6. COMPARISON OF CONTROL GROUP PARTICIPANTS WITH AND WITHOUT APP USAGE DATA 11](#_Toc198592760)

[Table S7. SUBGROUP ANALYSIS: TREATMENT EFFECTS AMONG MORE SEVERE SUBGROUP 12](#_Toc198592761)

[Table S8. SUBGROUP ANALYSIS: TREATMENT EFFECTS AMONG HIGHLY ENGAGED PARTICIPANTS 12](#_Toc198592762)

[Table S9. MIXED-EFFECTS AND GEE MODELS USING PHQ-A SCORES OVER TIME 13](#_Toc198592763)

[Table S10. ADVERSE EVENTS AMONGST PARTICIPANTS 14](#_Toc198592764)

[Table S11. RISK MANAGEMENT INTERVENTIONS 15](#_Toc198592765)

# **Table S1. KUAMSHA APP’S MAIN COMPONENTS**

| **App component** | **Description** | | **Screenshot** |
| --- | --- | --- | --- |
| Log-in unlock code | Kuamsha is password-protected. Users need to enter a password every time they access the app. | | 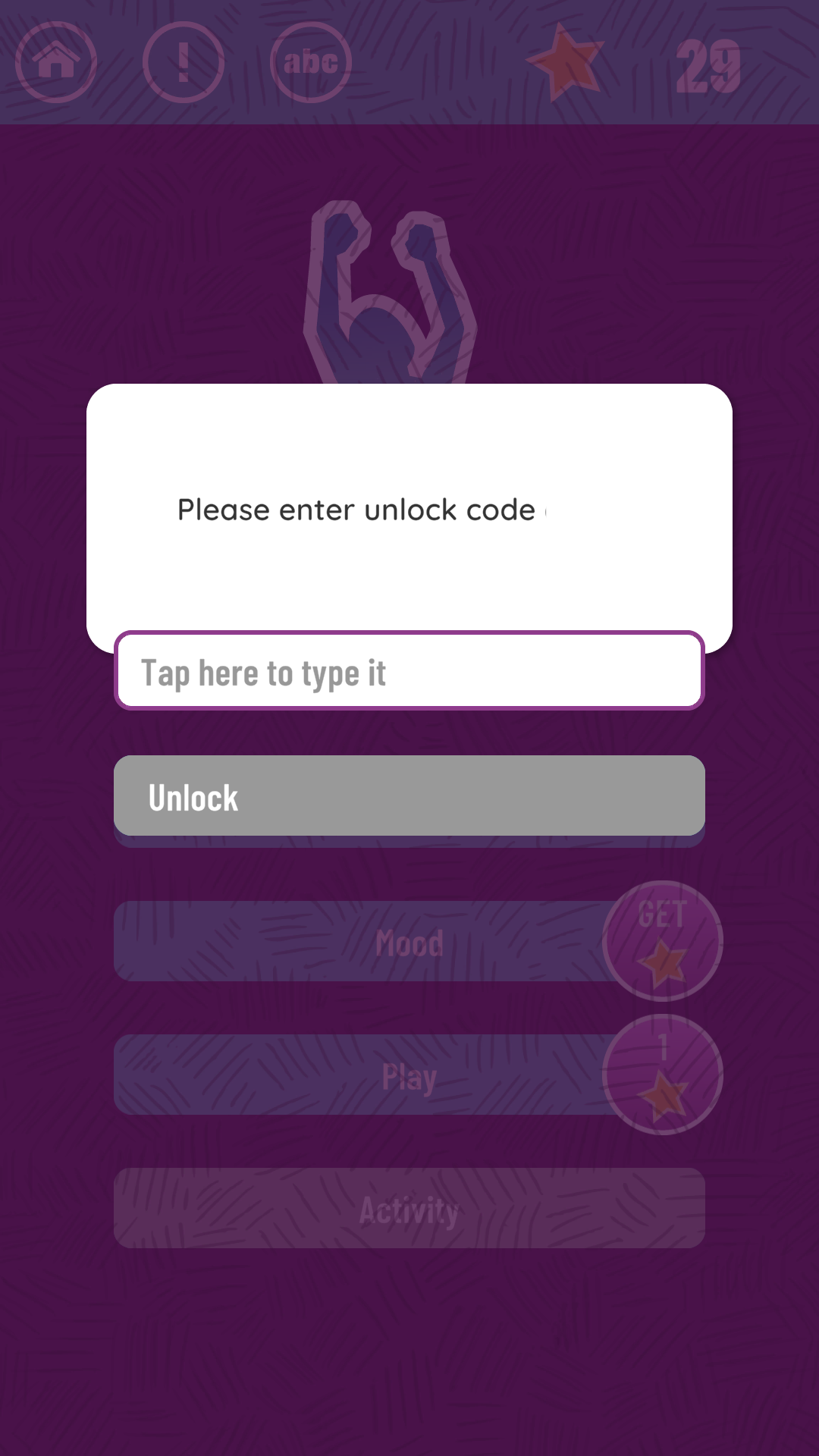 |
| Home screen | This is the first screen that users see as they log in to the Kuamsha app. Users have the option to play through the stories, monitor their mood, play absorbing activities to improve focus, or report on their weekly activities (see the rest of the table for further details on each of these components). | | 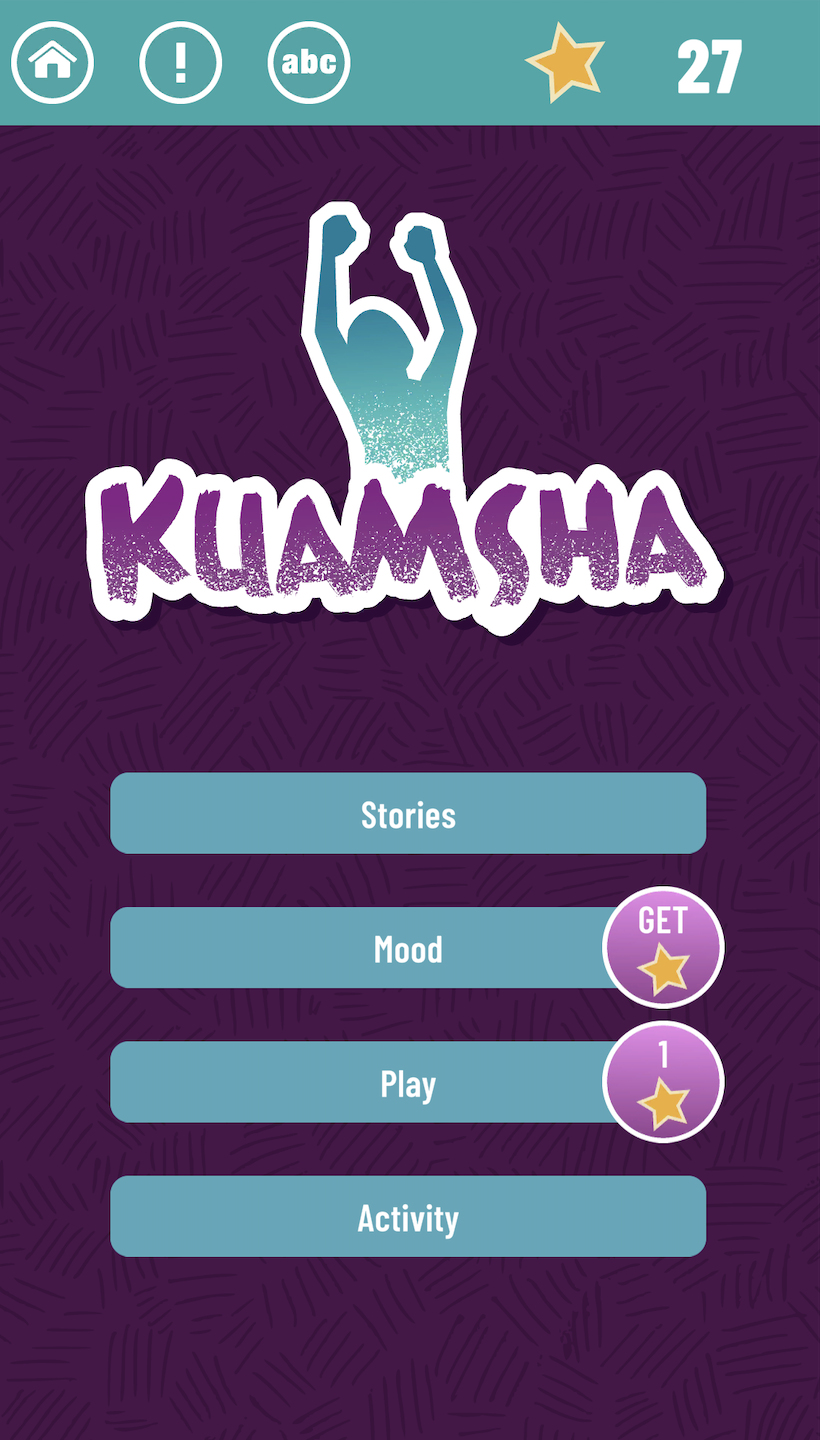 |
| Onboarding process | When users open the app for the first time, they are introduced and guided through its main components. This onboarding process aims to teach adolescents how to interact with the interface, choose their preferred language, locate the emergency button, and select one of the stories. | | 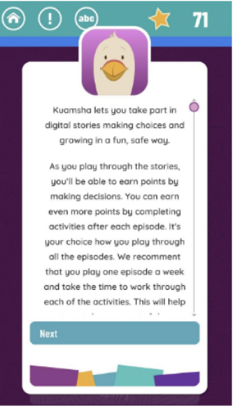 |
| Language selector | Users can select their preferred language. All the text on the app underwent 2 rounds of translation and has been checked by a clinical psychologist for accuracy. | | 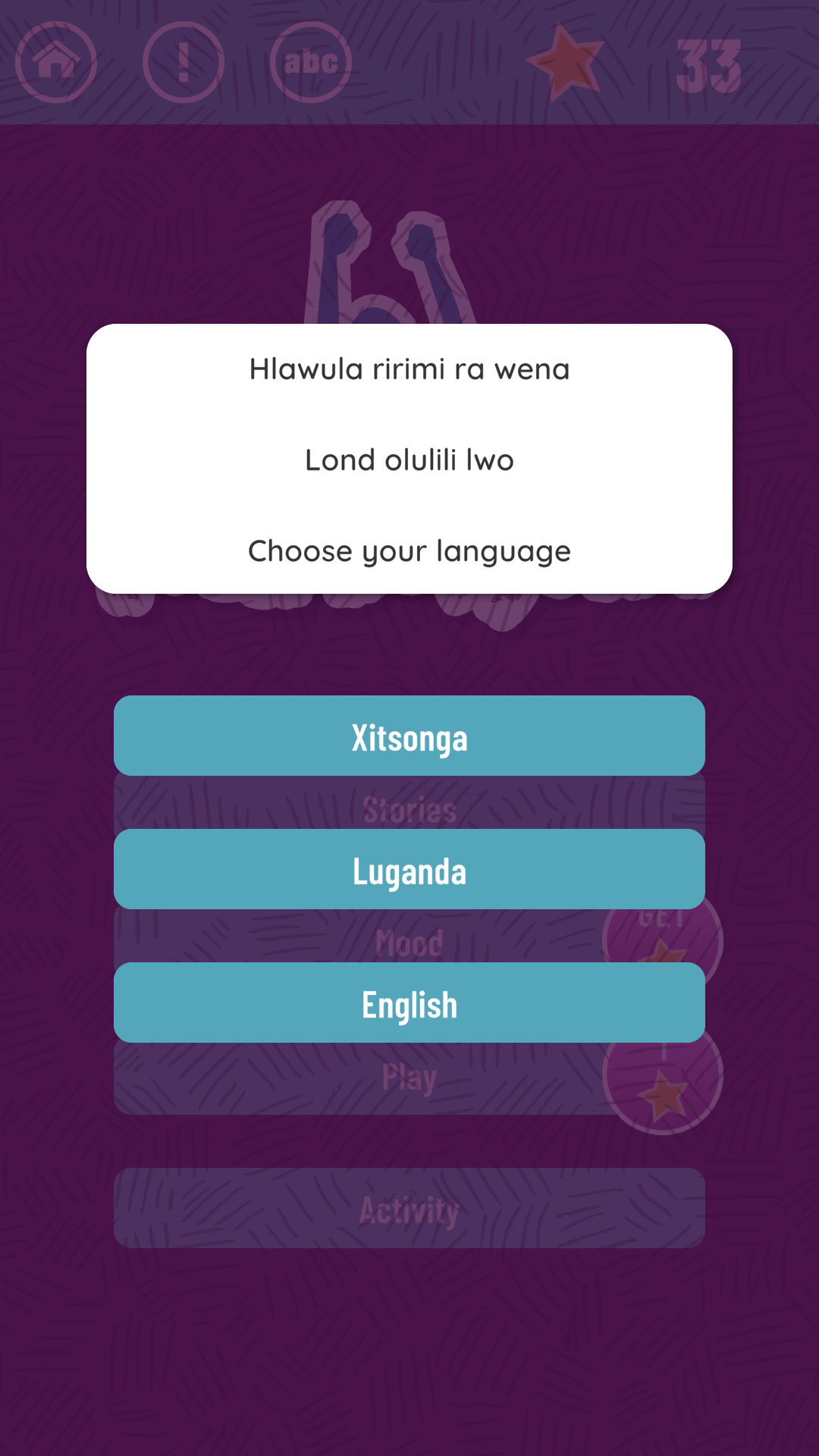 |
| Story selection | The core of the game consists of a choice between 2 narrative stories. Each consists of 6 modules that are played in sequential order. It is possible to begin one story and then switch to the other. During gameplay, points are earned for the choices made and by completing other core game elements described in this table. | | 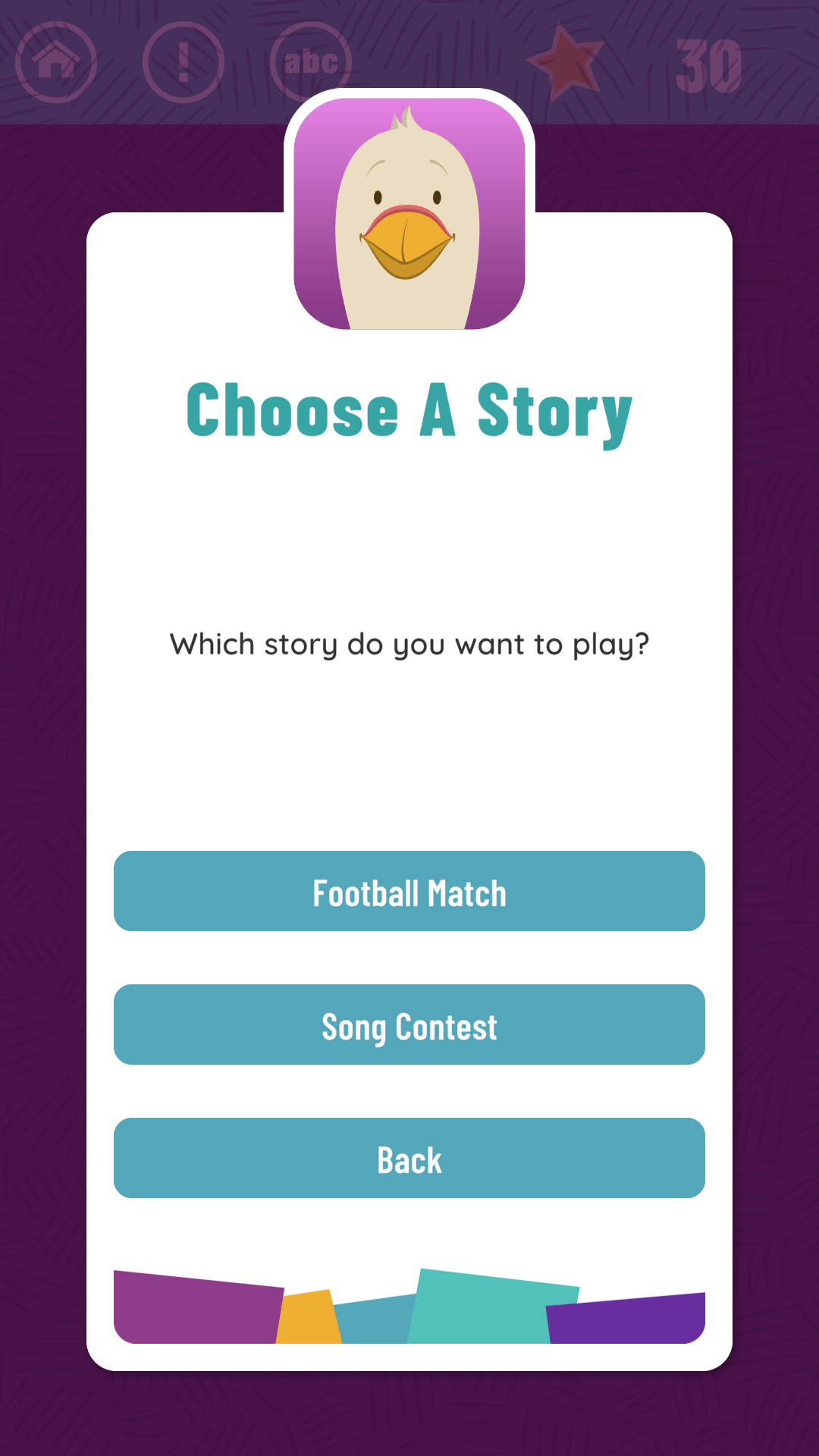 |
| Choices affect outcomes | A key principle of the game is that the player is drawn into the narrative through the use of decision points that allow users to shape the narrative. Choices made by the player branch the story in ways that lead the character down different pathways in the game. | | 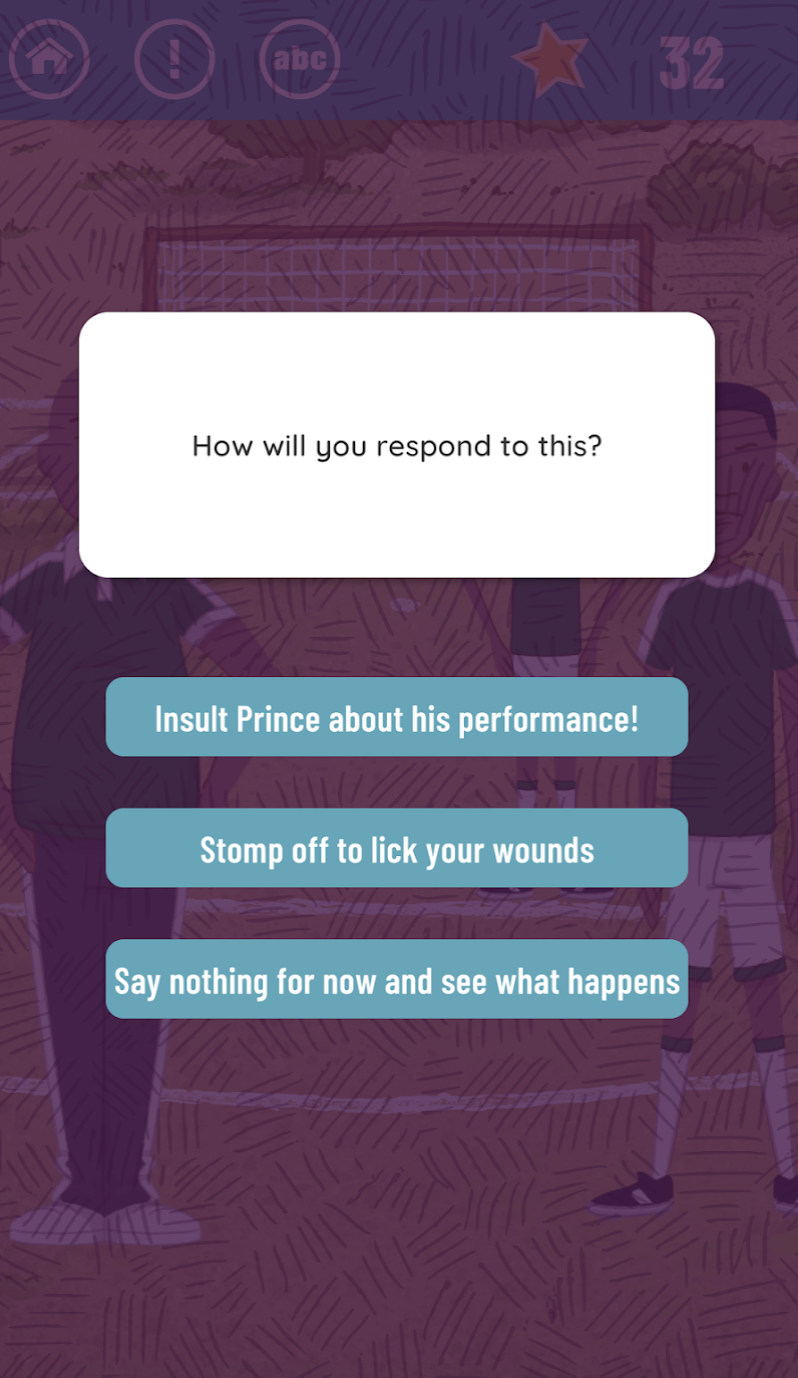 |
| Bird guide | The bird character is used throughout the game as a way of having players reflect on their choices, supporting review of what has been learned and offering suggestions and other game mechanics designed to draw the multiple components of the game into a unified whole. | | 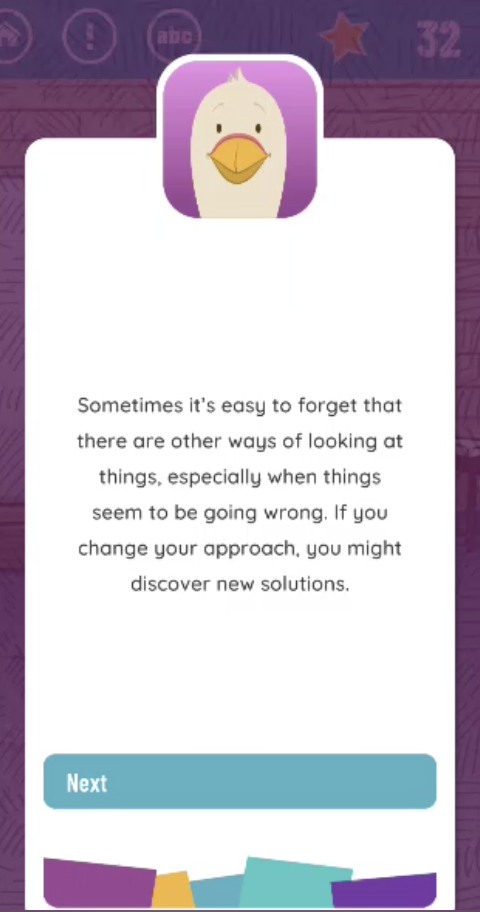 |
| Summary of lessons learned | To ensure that players are reflecting on the game and show some level of understanding of the concepts and choices made, each story episode ends with a summary of lessons learned. | | 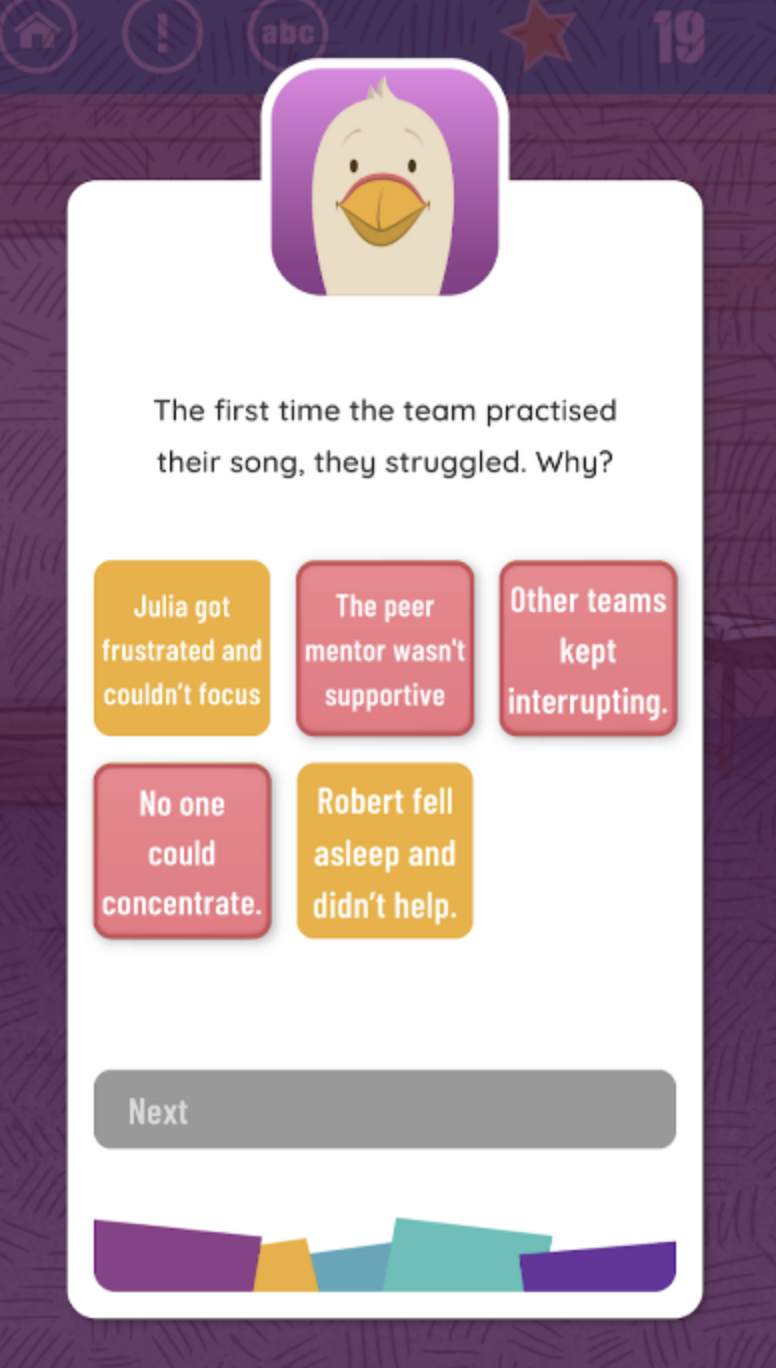 |
| Real-life exercises | Users are asked to think about an activity that they could do that is associated with the principle of the episode they just completed. Each homework session consists of 1 or 2 activities that expand on the behavioral activation principle covered in that session. | | 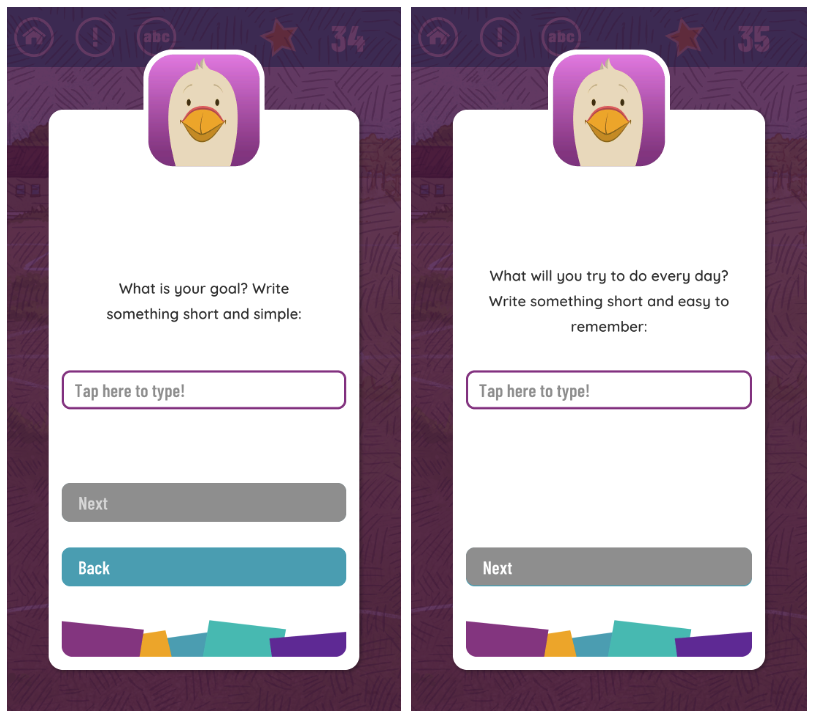 |
| Mood monitoring | Participants are asked to monitor their mood at different times (before and after the episode, when they report on homework, and when they complete an absorbing activity). Users receive feedback on how their mood changes over time and in-app points every time they complete the mood monitoring. | | 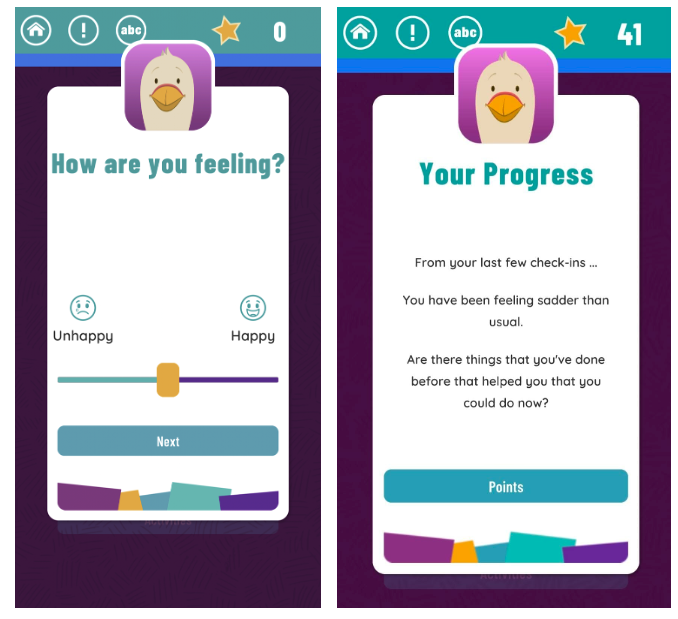 |
| Notifications | Users are reminded to report their progress on their weekly activities via notifications. | | 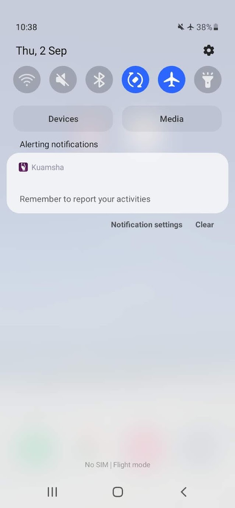 |
| Game design elements: personalization | Users are asked to personalize their character in the story by choosing their name, their preferred pronouns, and their team’s name. | | 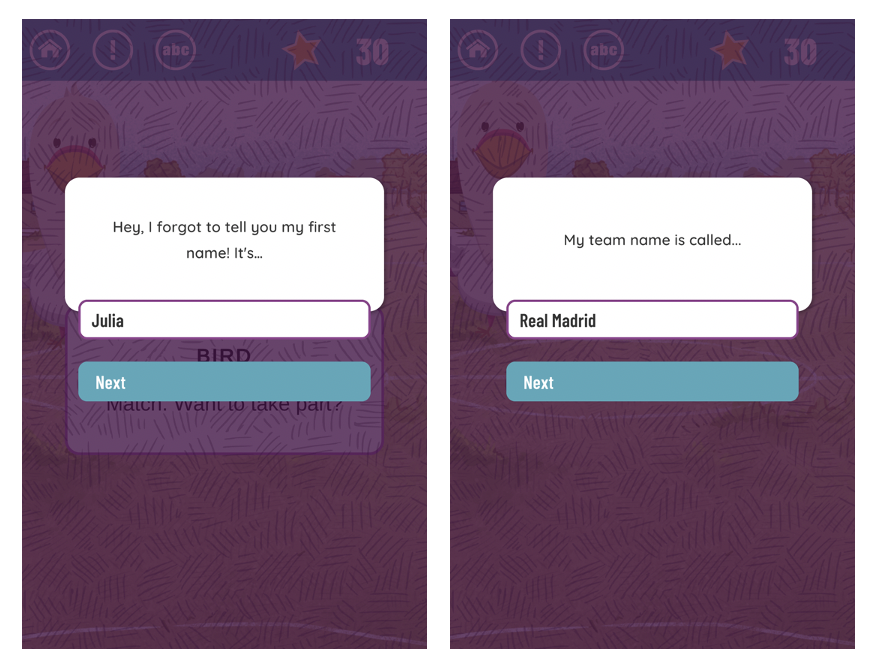 |
| Game design elements: in-app points | Participants earn in-app points every time they complete an episode, report their weekly activity, monitor their mood, and play the absorbing activities. | | 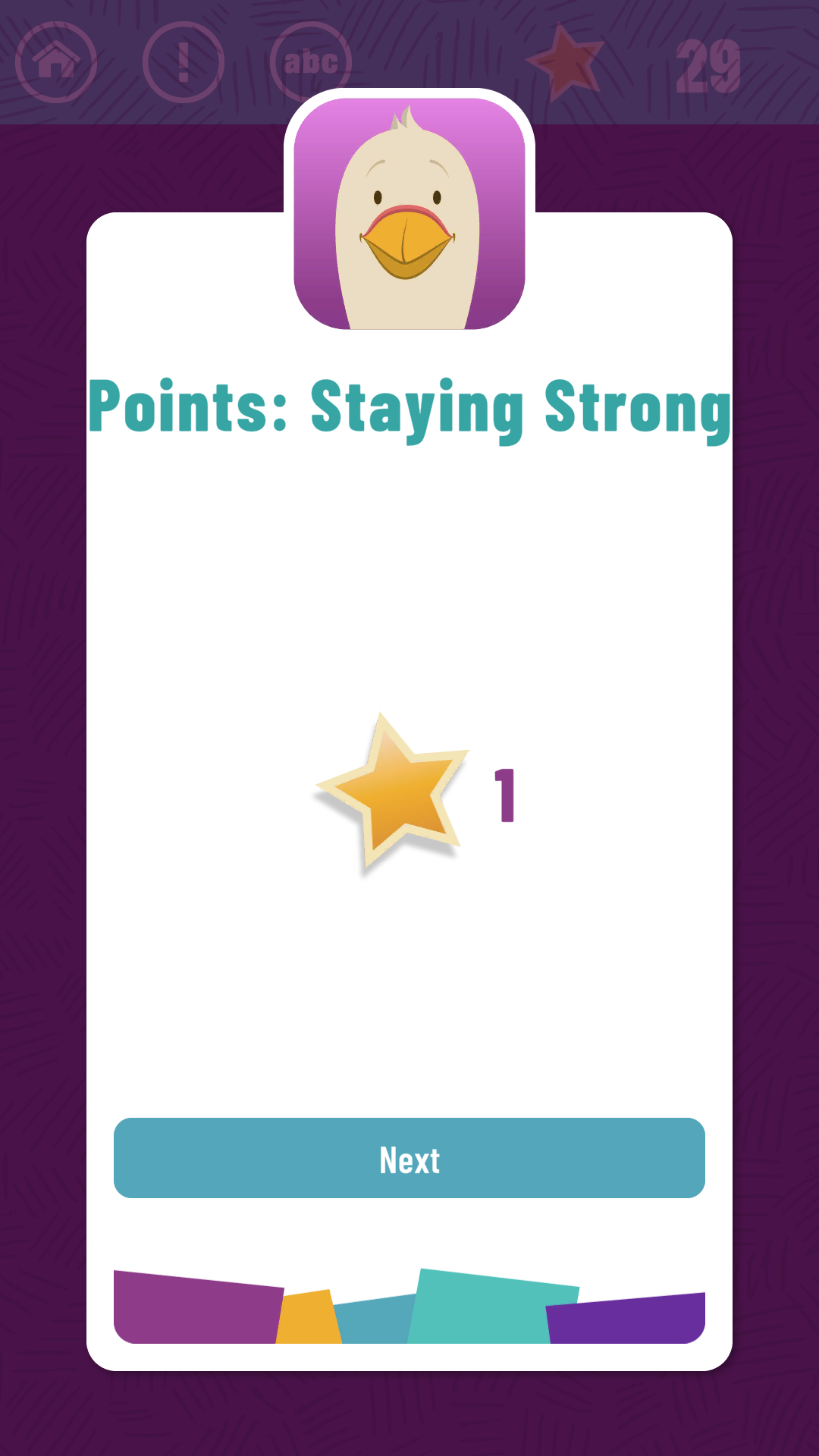 |
| Game design elements: absorbing activities | Kuamsha includes 2 different activities to teach the concept of absorption (focusing on an activity to detract from negative thoughts). Participants can decide between 2 different absorbing activities: a music absorbing activity (rhythm game in which users tap the screen in time with the music) and a football absorbing activity (users practice taking shots on goal). | | 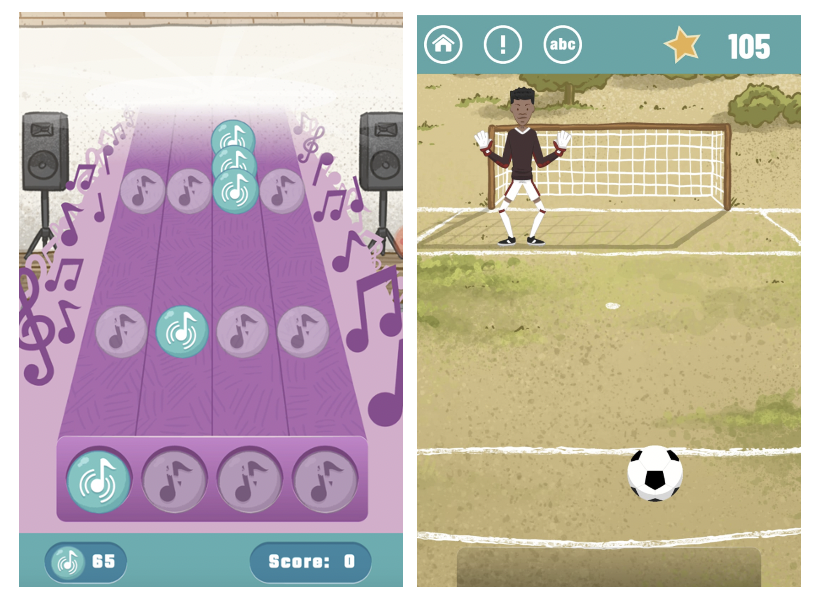 |
| Emergency button | Kuamsha includes an “emergency button” for adolescents to call in the event that they are feeling extremely sad or thinking about hurting themselves. | | 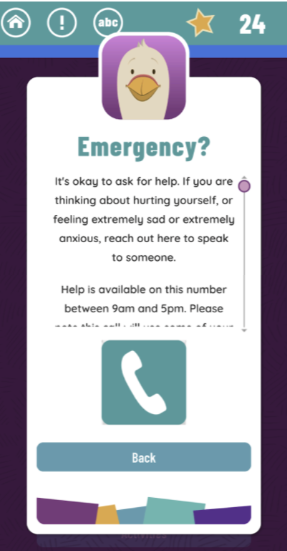 |
| Story characters | | 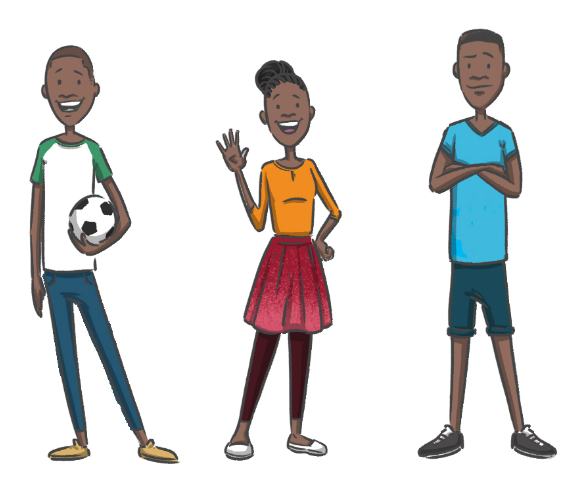 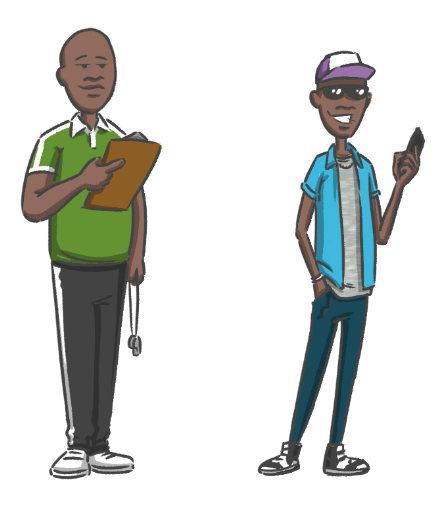 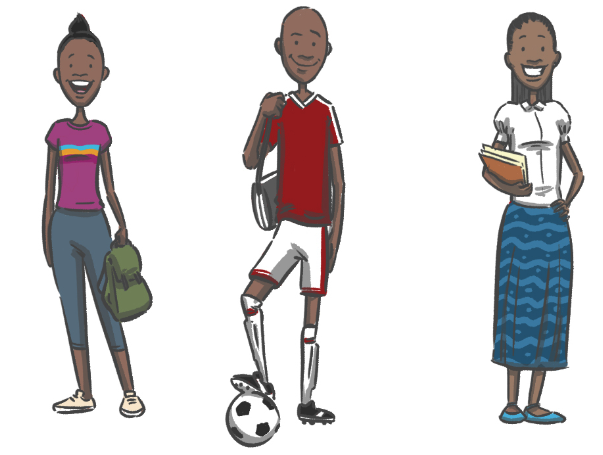 | |
| Background | | 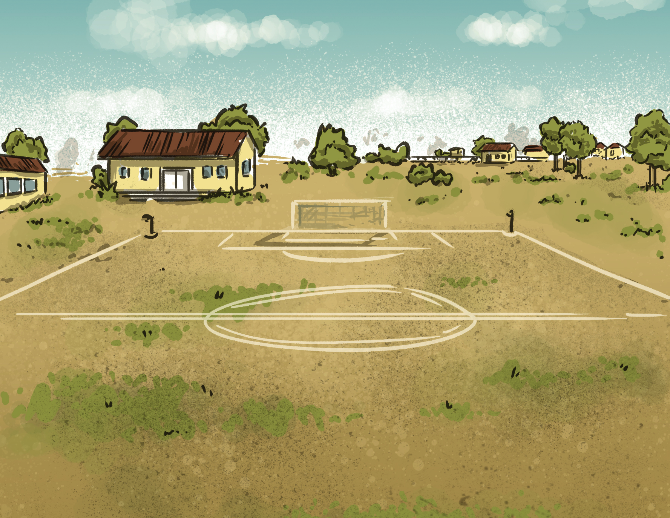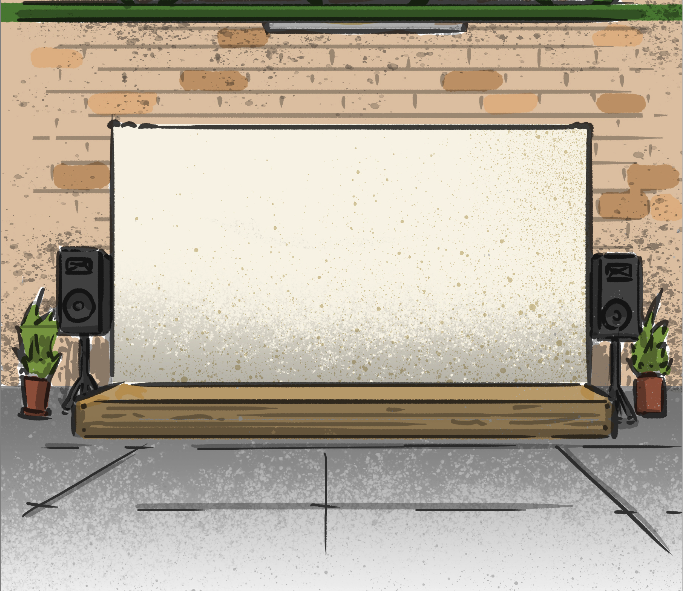  Football pitch Song contest stage  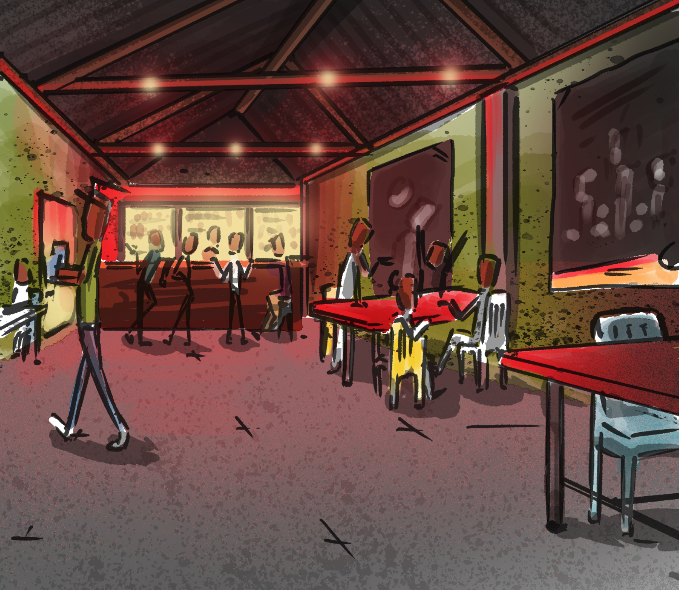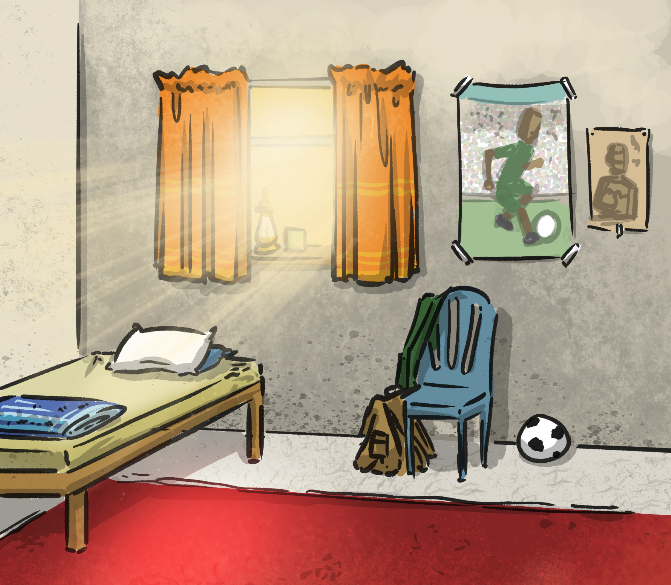  Tavern Main character’s room | |

# **Table S2. TRIAL PROGRESSION CRITERIA**

| **Criterion** | **Pre-determined cut-off** | | | **Results from the DoBat study** |
| --- | --- | --- | --- | --- |
|  | **Green** | **Amber** | **Red** |  |
| **Enrolment** (proportion of eligible participants enrolled) | ≥60% | <60%, ≥40% | <40% | 97% |
| **Retention** to trial at 11 weeks | ≥90% | <90%, ≥50% | <50% | 100% |
| **Treatment adherence:** Proportion of participants that opened at least 4 app episodes | ≥70% | <70%, ≥50% | <50% | 81.25% |
| **Treatment adherence:** Proportion of participants that had at least 3 phone calls with the peer mentor | ≥70% | <70%, ≥50% | <50% | 78.7% |

# **Table S3. CONSORT EXTENSION FOR PILOT AND FEASIBILITY STUDIES CHECKLIST**

| Section/Topic | Item No | Checklist item | Reported on page No |
| --- | --- | --- | --- |
| Title and abstract | | | |
|  | 1a | Identification as a pilot or feasibility randomised trial in the title | 1 |
|  | 1b | Structured summary of pilot trial design, methods, results, and conclusions (for specific guidance see CONSORT abstract extension for pilot trials) | 1 |
| Introduction | | | |
| Background and objectives | 2a | Scientific background and explanation of rationale for future definitive trial, and reasons for randomised pilot trial | 2-4 |
|  | 2b | Specific objectives or research questions for pilot trial | 4 |
| Methods | | | |
| Trial design | 3a | Description of pilot trial design (such as parallel, factorial) including allocation ratio | 4 |
|  | 3b | Important changes to methods after pilot trial commencement (such as eligibility criteria), with reasons | n/a |
| Participants | 4a | Eligibility criteria for participants | 4 |
|  | 4b | Settings and locations where the data were collected | 6 |
|  | 4c | How participants were identified and consented | Study protocol |
| Interventions | 5 | The interventions for each group with sufficient details to allow replication, including how and when they were actually administered | 8 and Table S1 |
| Outcomes | 6a | Completely defined prespecified assessments or measurements to address each pilot trial objective specified in 2b, including how and when they were assessed | 6-7 |
|  | 6b | Any changes to pilot trial assessments or measurements after the pilot trial commenced, with reasons | n/a |
|  | 6c | If applicable, prespecified criteria used to judge whether, or how, to proceed with future definitive trial | 6-7 and Table S2 |
| Sample size | 7a | Rationale for numbers in the pilot trial | 7 |
|  | 7b | When applicable, explanation of any interim analyses and stopping guidelines | n/a |
| Randomisation: |  |  |  |
| Sequence  generation | 8a | Method used to generate the random allocation sequence | 4-5 |
|  | 8b | Type of randomisation(s); details of any restriction (such as blocking and block size) | 4-5 |
| Allocation  concealment  mechanism | 9 | Mechanism used to implement the random allocation sequence (such as sequentially numbered containers), describing any steps taken to conceal the sequence until interventions were assigned | n/a |
| Implementation | 10 | Who generated the random allocation sequence, who enrolled participants, and who assigned participants to interventions | 4-5 |
| Blinding | 11a | If done, who was blinded after assignment to interventions (for example, participants, care providers, those assessing outcomes) and how | 4-5 |
|  | 11b | If relevant, description of the similarity of interventions | n/a |
| Statistical methods | 12 | Methods used to address each pilot trial objective whether qualitative or quantitative | 7-8 |
| Results | | | |
| Participant flow (a diagram is strongly recommended) | 13a | For each group, the numbers of participants who were approached and/or assessed for eligibility, randomly assigned, received intended treatment, and were assessed for each objective | Figure 1 |
|  | 13b | For each group, losses and exclusions after randomisation, together with reasons | Figure 1 |
| Recruitment | 14a | Dates defining the periods of recruitment and follow-up | 8 |
|  | 14b | Why the pilot trial ended or was stopped | n/a |
| Baseline data | 15 | A table showing baseline demographic and clinical characteristics for each group | Table 1 |
| Numbers analysed | 16 | For each objective, number of participants (denominator) included in each analysis. If relevant, these numbers  should be by randomised group | Tables 2 & 3 |
| Outcomes and estimation | 17 | For each objective, results including expressions of uncertainty (such as 95% confidence interval) for any  estimates. If relevant, these results should be by randomised group | Tables 2 & 3 |
| Ancillary analyses | 18 | Results of any other analyses performed that could be used to inform the future definitive trial | 11 |
| Harms | 19 | All important harms or unintended effects in each group (for specific guidance see CONSORT for harms) | Table S10 |
|  | 19a | If relevant, other important unintended consequences | n/a |
| Discussion | | | |
| Limitations | 20 | Pilot trial limitations, addressing sources of potential bias and remaining uncertainty about feasibility | 12-14 |
| Generalisability | 21 | Generalisability (applicability) of pilot trial methods and findings to future definitive trial and other studies | 12-14 |
| Interpretation | 22 | Interpretation consistent with pilot trial objectives and findings, balancing potential benefits and harms, and  considering other relevant evidence | 14 |
|  | 22a | Implications for progression from pilot to future definitive trial, including any proposed amendments | 14 |
| Other information | | |  |
| Registration | 23 | Registration number for pilot trial and name of trial registry | PACTR202206574814636 |
| Protocol | 24 | Where the pilot trial protocol can be accessed, if available | 4, Methods |
| Funding | 25 | Sources of funding and other support (such as supply of drugs), role of funders | Title page |
|  | 26 | Ethical approval or approval by research review committee, confirmed with reference number | 4, Methods |

Citation: Eldridge SM, Chan CL, Campbell MJ, Bond CM, Hopewell S, Thabane L, et al. CONSORT 2010 statement: extension to randomised pilot and feasibility trials. BMJ. 2016;355. This is an Open Access article distributed in accordance with the terms of the Creative Commons Attribution (CC BY 3.0) license (<http://creativecommons.org/licenses/by/3.0/>), which permits others to distribute, remix, adapt and build upon this work, for commercial use, provided the original work is properly cited.

# **Table S4. COMPLETION RATES OF STUDY ASSESSMENTS**

|  | **Total** | **Control** | **Treatment** | **P-value** |
| --- | --- | --- | --- | --- |
| **Study assessment** | **N=195** | **N=99** | **N=96** |  |
| Screening date | 100% | 100% | 100% |  |
| Baseline date | 100% | 100% | 100% |  |
| Symptom monitoring: Week 2.5 | 45·6% | 55·6% | 35·4% | 0.005 |
| Symptom monitoring: Week 5 | 43·6% | 47·5% | 39·6% | 0.27 |
| Symptom monitoring: Week 7.5 | 46·2% | 54·5% | 37·5% | 0.017 |
| Week 11 | 100% | 100% | 100% |  |
| Acceptability questionnaire | 99·5% | 100.0% | 99·0% | 0.31 |
| Week 24 | 98·5% | 97·0% | 100% | 0.086 |

# **Table S5. FEASIBILITY OUTCOMES BY DEPRESSION STATUS**

| **TREATMENT GROUP** | **Non-severe**  **(n=67)** | **Severe**  **(n=29)** | **P-value** |
| --- | --- | --- | --- |
|  |  |  |  |
| Number of participants with data, n(%) | 67 (100) | 29 (100) |  |
|  |  |  |  |
| **Treatment adherence** |  |  |  |
| Opened ≥4 app modules, n(%) | 56 (83·58) | 21 (72·41) | 0·211 |
| Had ≥3 phone calls with peer mentors, n(%) | 53 (79·10) | 24 (82·76) | 0·684 |
|  |  |  |  |
| **Kuamsha app engagement metrics** |  |  |  |
| Log ins, average (SD) | 32·24 (33·55) | 23·76 (19·62) | 0·208 |
| Modules opened, average (SD) | 16·96 (18·64) | 14·76 (14·57) | 0·574 |
| Modules completed, average (SD) | 12·55 (15·49) | 11.41 (12·92) | 0·730 |
| Total time spent on app (hours:min), average (SD) | 03:53 (04:16) | 2:53 (02:43) | 0·262 |
| Set up weekly activities, average (SD) | 5·76 (4·09) | 5·31 (4·89) | 0·642 |
| Completed weekly activities, average (SD) | 50·06 (91·13) | 69·48 (183) | 0·489 |
|  |  |  |  |
| **Peer mentor engagement metrics**  *(total of 512 calls)* |  |  |  |
| Phone call duration (min), average (SD) | 16·58 (4·85) | 16·59 (4·85) | 0·977 |
| Time days between phone calls, average (SD) | 9·86 (9·33) | 9·71 (7·45) | 0·876 |
| Participant who completed all phone calls, n(%) | 33 (50·77) | 17 (58·62) | 0·486 |
|  |  |  |  |
| **CONTROL GROUP** | **Non-severe**  **(N=69)** | **Severe**  **(N=30)** | **P-value** |
| Number of participants with data, n(%) | 43 (62) | 18 (60) |  |
|  |  |  |  |
| Videos opened, average (SD) | 2·56 (1·86) | 2·06 (1·80) | 0·334 |
| Videos completed, average (SD) | 1·05 (1·45) | 0·89 (1·53) | 0·704 |
| Total time spent on app (hours:min), average (SD) | 00:28 (00:35) | 00:22 (00:33) | 0.565 |

#

# **Table S6. COMPARISON OF CONTROL GROUP PARTICIPANTS WITH AND WITHOUT APP USAGE DATA**

|  | **Control total**  **(n=99)** | **Without data**  **(n=38)** | **With data**  **(n=61)** | **P-value** |
| --- | --- | --- | --- | --- |
| Age, mean (SD) | 16·26 (1·14) | 16·26 (1·22) | 16·26 (1·09) | 1.00 |
| Gender |  |  |  | 0.012 |
| Male (%) | 32·32 | 47·37 | 22·95 |  |
| Female (%) | 67·68 | 52·63 | 77·05 |  |
| Married (%) | 5·05 | 7·89 | 3·28 | 0.31 |
| Lost a parent (%) | 35·35 | 39·47 | 32·79 | 0.50 |
| Has children (%) | 7·87 | 8·33 | 7·55 | 0.89 |
| Grade enrolled at screening |  |  |  | 0.19 |
| Grade 8 (%) | 1·01 | 2·63 | 0·00 |  |
| Grade 9 (%) | 10·10 | 10·53 | 9·84 |  |
| Grade 10 (%) | 40·40 | 50·00 | 34·43 |  |
| Grade 11 (%) | 48·48 | 36·84 | 55·74 |  |
| Did any work past 7 days (%) | 20·62 | 23·68 | 18·64 | 0.55 |
| Household asset index, mean (SD) | 41·72 (14·94) | 40·84 (12·99) | 42·27 (16·13) | 0.65 |
| Food insecure (%) | 71·88 | 64·86 | 76·27% | 0.23 |
| Caregiver's years of education, mean (SD) | 8·59 (4·54) | 8·03 (5·38) | 8·89 (4·03) | 0.41 |
| PHQ-A score, mean (SD) | 8·40 (3·55) | 8·34 (3·60) | 8·44 (3·55) | 0.89 |
| Depression symptom category |  |  |  | 0.87 |
| Mild (5≤PHQ-A<10) | 69·70 | 68·42 | 70·49 |  |
| Moderate (10≤PHQ-A<15) | 21·21 | 23·68 | 19·67 |  |
| Moderately severe (15≤PHQ-A<19) | 9·09 | 7·89 | 9·84 |  |
| Data are n (%), mean (SD), or n/N (%). **Gender** is based on self-report. **Caregiver** refers to an adult with joint or sole caring responsibility in the index adolescent’s household. **PHQ-A** = 9-item Patient Health Questionnaire- Adolescent Version. **Household asset index** was measured using the Simple Poverty Scorecard Poverty-Assessment Tool South Africa.^33^ For reference, scores between 38-40 correspond to a 9.8% likelihood of living below the international extreme poverty line of $1·90/day (PPP, 2011 prices). **Food insecurity** was measured using the 6-item Food Security Module.^34^ | | | |  |

# **Table S7. SUBGROUP ANALYSIS: TREATMENT EFFECTS AMONG MORE SEVERE SUBGROUP**

The table below shows the treatment effect among the participants who scored≥10 in the PHQ-A at screening.

|  | **Treatment group**  **(N=29)** | **Control group**  **(N=30)** | **Cohen’s d**  **(95% CI)** | **Adjusted mean difference**  **(95% CI)** | **p-value** |
| --- | --- | --- | --- | --- | --- |
| Mean PHQ-A score at 11 weeks (SD) for participants with PHQ-A score≥10 at screening^a^ | 8·66 (6·15) | 8·7 (6·00) | -0.07 (-0·59, 0·46) | -0·40 (-3·56, 2·75) | 0·798 |
| Mean PHQ-A score at 24 weeks (SD) for participants with PHQ-A score≥10 at screening^a^ | 7·76 (5·90) | 9·17 (5·39) | -0.30 (-0·88, 0·28) | -1·62 (-4·76, 1·51) | 0·304 |

^a^ Linear regression adjusted for age, sex, Household Assets Index, and PHQ-A score at screening. We used robust standard errors to allow for the presence of heteroskedasticity.

# **Table S8. SUBGROUP ANALYSIS: TREATMENT EFFECTS AMONG HIGHLY ENGAGED PARTICIPANTS**

The table below shows the treatment effect among the highly engaged participants (defined as those in the top third in terms of their app usage time).

|  | **Treatment group**  **(n=32)** | **Control group**  **(n=20)** | **Cohen’s *d***  **(95% CI)** | **Adjusted mean difference**  **(95% CI)** | **p-value** |
| --- | --- | --- | --- | --- | --- |
| Mean PHQ-A score at 11 weeks (SD) for highly engaged participants^a^ | 6·19 (4·47) | 7·6 (5·73) | -0.26 (-0·78, 0·27) | -1·47 (-4·48, 1·53) | 0·328 |
| Mean PHQ-A score at 24 weeks (SD) for highly engaged participants^a^ | 7·13 (4·67) | 8·25 (6·90) | -0.18 (-0·71, 0·34) | -1·26 (-4·91, 2·38) | 0·489 |

^a^ Linear regression adjusted for age, sex, Household Assets Index, and PHQ-A score at screening. We used robust standard errors to allow for the presence of heteroskedasticity.

# **Table S9. MIXED-EFFECTS AND GEE MODELS USING PHQ-A SCORES OVER TIME**

|  | **PHQ models fit** |  |
| --- | --- | --- |
| Variable (Categories) | **Mixed effects model** | **GEE model** |
| **Trial (Arm A – ref)** |  |  |
| Arm B | 0.189 | 0.0169 |
|  | (0.474) | (0.542) |
| **Time (Screening -ref)** |  |  |
| 1.Week 2.5 | -2.194*** | -2.223*** |
|  | (0.495) | (0.487) |
| 2. Week 5.0 | -1.700*** | -1.691*** |
|  | (0.453) | (0.452) |
| 3.Week 7.5 | -2.095*** | -2.045*** |
|  | (0.492) | (0.488) |
| 4.Week 11 (Primary endpoint) | -1.225*** | -1.225*** |
|  | (0.428) | (0.428) |
| 5.Week 24 (Secondary endpoint) | -1.141*** | -1.141*** |
|  | (0.433) | (0.433) |
| **Gender (Male – ref)** |  |  |
| Female | 0.742 | 1.237** |
|  | (0.470) | (0.536) |
| **Household Assets Index** | 0.0382** | 0.0306 |
|  | (0.0158) | (0.0195) |
| **Age** | 0.212 | 0.157 |
|  | (0.208) | (0.247) |
| **Constant** | 2.997 | 3.937 |
|  | (3.485) | (4.139) |
|  |  |  |
| Observations | 843 | 843 |
| Number of groups | 191 |  |
| Number of id |  | 191 |

Robust standard errors in parentheses

*** p<0.01, ** p<0.05, * p<0.1

# **Table S10. ADVERSE EVENTS AMONGST PARTICIPANTS**

| **Adverse events** | **Screened Participants**  **(n= 842)** | **Enrolled Participants**  **(n=195)** | | |
| --- | --- | --- | --- | --- |
|  |  | **Treatment Group (n=96)** | **Control Group**  **(n=99)** | **RD (95% CI)** |
| Died by suicide | 0 (0.00) | 0 (0,00) | 1 (1,01) | -1,01 (-2.93 to 5.50) |
| Suicide attempt | 0 (0.00) | 0 (0,00) | 1 (1,01) | -1,01 (-2.93 to 5.50) |
| Suicidal ideation (total) | 41 (4.87) | 19 (19,79) | 10 (10,10) | 9.69 (-0.39 to 19.82) |
| High Risk | 6 (0.71) | 2 (2,08) | 1 (1,01) | 1,07 (-3.67 to 6.33) |
| Moderate Risk | 12 (1.43) | 8 (8,33) | 5 (5,05) | 3,28 (-4.15 to 11.08) |
| Low Risk | 23 (2.73) | 9 (9,38) | 4 (4,04) | 5,34 (-1.99 to 13.22) |
| Severe depression | 7 (0.83) | 3 (3.13) | 2 (2.02) | 1.11 (-4.34 to 6.96) |
| High risk substance abuse | 0 (0.00) | 0 (0,00) | 3 (3,03) | -3,03 (-1.30 to 8.53) |
| Physical assault | 7 (0.83) | 1 (1,04) | 3 (3,03) | -1,99 (-3.05 to 7.56) |
| Sexual assault | 5 (0.59) | 0 (0,00) | 1 (1,01) | -1,01 (-2.93 to 5.50) |
| Bullying | 3 (0.36) | 1 (1,04) | 0 (0,00) | 1,04 (-2.79 to 5.66) |
| Food Insecurity | 4 (0.48) | 0 (0,00) | 1 (1,010 | -1,01 (-2.93 to 5.50) |
| Caregiver abuse/ neglect | 3 (0.36) | 1 (1,04) | 0 (0,00) | 1,04 (-2.79 to 5.66) |
| Orphan | 1 (0.12) | 0 (0,00) | 1 (1,01) | -1,01 (-2.93 to 5.50) |
| Data are n(%) or **Risk Difference (RD**) and 95% Confidence Interval. Risk Difference is the preferred statistical measure to assess difference in risks when an event is rare. Adverse events reported here include i) those that were **systematically assessed** based on participant responses to the Patient Health Questionnaire Adolescent Version (PHQ-A) conducted at screening; 2.5; 7.5; 11; 24weeks, as well as ii) those that were **spontaneously reported** to the study team. All screened and enrolled participants who had PHQ-A score >19 or PHQ-A 9^th^ item score ≥1 received a telephonic risk assessment by the Trial Registered Counsellor. Assessment and classification of suicidal risk was done according to a pre-defined Risk Management Protocol, based on the **Mini International Neuropsychiatric Interview (MINI)** suicide module. Participants who had a PHQ-A item 9 response ≥ 1 on the survey but indicated no thoughts of suicide or self-harm within the past month on the telephone call with the Registered Counsellor were classified as having “no current suicidal ideation” and their data is not included here. All adverse events reported here are considered incidental and not related to participation in the study. | | | | |

# **Table S11. RISK MANAGEMENT INTERVENTIONS**

| **Risk Management Interventions** | **Screened Participants**  **(n= 842)** | **Enrolled Participants**  **(n=195)** | | | **Post-trial Care**  **(n=195)** | | |
| --- | --- | --- | --- | --- | --- | --- | --- |
|  |  | **Treatment Group (n=96)** | **Control Group**  **(n=99)** | **RD (95% CI)** | **Treatment Group (n=96)** | **Control Group**  **(n=99)** | **RD (95% CI)** |
| **Risk Assessments (total)** | 46 (5.46) | 7 (7.07) | 6 (6.06) | 1.01 (-6.47 to 8.68) | 10 (10.42) | 8 (8.08) | 2.34 (-6.12 to 10.99) |
| Telephonic Assessment | 46 (5.46) | 7 (7.07) | 6 (6.06) | 1.01 (-6.47 to 8.68) | 10 (10.42) | 8 (8.08) | 2.34 (-6.12 to 10.99) |
| Home Visit | 12 (1.43) | 1 (1.01) | 1 (1.01) | 0.00 (-4.68 to 4.57) | 1 (1.04) | 1 (1.01) | 0.03 (-4.54 to 4.73) |
| **Referrals (total)** | 25 (2.97) | 6 (6.25) | 7 (7.07) | 0.82 (-6.81 to 8.41) | 12 (12.50) | 9 (9.09) | 3.41 (-5.55 to 12.53) |
| **Psychologist** |  |  |  |  |  |  |  |
| Attempted referrals | 12 (1.43) | 4 (4.04) | 1 (1.01) | 3.03 (-2.10 to 9.10) | 4 (4.17) | 3 (3.03) | 1.14 (-4.91 to 7.52) |
| Declined referrals | 2 (0.24) | 0 (0.00) | 0 (0.00) | 0.00 (-3.84 to 3.74) | 0 (0.00) | 0 (0.00) | 0.00 (-3.84 to 3.74) |
| Attended Appointments | 10 (1.19) | 4 (4.04) | 1 (1.01) | 3.03 (-2.10 to 9.10) | 4 (4.17) | 3 (3.03) | 1.14 (-4.91 to 7.52) |
| **Social Worker** |  |  |  |  |  |  |  |
| Attempted referrals | 13 (1.54) | 2 (2.02) | 6 (6.06) | 4.04 (-2.07 to 10.74) | 0 (0.00) | 1 (1.01) | 1.01 (-2.93 to 5.50) |
| Declined referrals | 4 (0.48) | 0 (0.00) | 0 (0.00) | 0.00 (-3.84 to 3.74) | 0 (0.00) | 0 (0.00) | 0.00 (-3.84 to 3.74) |
| Attended Appointments | 9 (1.07) | 2 (2.02) | 6 (6.06) | 4.04 (-2.07 to 10.74) | 0 (0.00) | 1 (1.01) | 1.01 (-2.93 to 5.50) |
| **Medical Officer** | 1 (0.12) | 0 (0.00) | 0 (0.00) | 0.00 (-3.84 to 3.74) | 3 (3.13) | 1 (1.01) | 2.12 (-2.82 to 7.85) |
| **GRIP** | 3 (0.36) | 0 (0.00) | 0 (0.00) | 0.00 (-3.84 to 3.74) | 0 (0.00) | 0 (0.00) | 0.00 (-3.84 to 3.74) |
| **COGTA** | 2 (0.24) | 0 (0.00) | 1 (1.01) | 1.01 (-2.92 to 5.50) | 0 (0.00) | 0 (0.00) | 0.00 (-3.84 to 3.74) |
| **Clinic-based Registered Counsellor** | n/a | n/a | n/a | n/a | 5 (5.21) | 4 | 1.17 (-5.43 to 8.04) |
| **Antidepressant initiated** | 1 (0.12) | 0 (0.00) | 0 (0.00) | 0.00 (-3.84 to 3.74) | 3 (3.13) | 1 (1.01) | 2.12 (-2.82 to 7.85) |
| Data are n (%) or **Risk Difference (RD**) and 95% Confidence Interval. Risk Difference is the preferred statistical measure to assess difference in risks when an event is rare. **Risk management interventions are divided into the following categories: i) Screened participants** = interventions provided to all participants who completed the screening survey (including participants who were not eligible to be enrolled in the trial) ii) **Enrolled participants** = interventions provided to participants enrolled in the trial between enrollment and 24-weeks iii) **Post-trial care** = interventions provided to enrolled participants after they completed the 24-week follow-up survey. **Transport** was provided for all appointments at the local district hospital (i.e. to see the psychologist or medical officer). A **Registered Counsellor** was appointed to the local clinic at the end of the study, therefore participants could only be referred to the counsellor for post-trial care. **Antidepressant** medication was initiated by medical officers at the local district hospital based on their assessment of major depressive disorder. There are no psychiatrists or child and adolescent psychiatrists working in the government sector in Bushbuckridge.**GRIP** = Greater Rape Intervention Programme. **COGTA** = Department of Cooperative Governance and Traditional Affairs. | | | | | | | |
